# *In situ* small-angle X-ray scattering studies of sterically-stabilized diblock copolymer nanoparticles formed during polymerization-induced self-assembly in non-polar media†
†Electronic supplementary information (ESI) available: Experimental details including *in situ* SAXS measurements; synthesis and characterization of (co)polymers; kinetic study for the synthesis of a PSMA_31_ macro-CTA; data modelling for *in situ* SAXS experiments including kinetic renormalization, determination of BzMA monomer within the nanoparticle cores and estimation of the standard deviation in the molecular weight distribution; example 2D SAXS patterns; TEM images of octopi and jellyfish; DLS, TEM and SAXS analyses of PSMA_13_–PBzMA_*x*_ vesicles; SAXS models used for spherical micelles and vesicles. See DOI: 10.1039/c6sc01243d


**DOI:** 10.1039/c6sc01243d

**Published:** 2016-04-18

**Authors:** Matthew J. Derry, Lee A. Fielding, Nicholas J. Warren, Charlotte J. Mable, Andrew J. Smith, Oleksandr O. Mykhaylyk, Steven P. Armes

**Affiliations:** a Department of Chemistry , The University of Sheffield , Dainton Building, Brook Hill , Sheffield , South Yorkshire S3 7HF , UK . Email: s.p.armes@sheffield.ac.uk ; Email: o.mykhaylyk@sheffield.ac.uk ; Email: m.derry@sheffield.ac.uk; b Diamond Light Source Ltd , Diamond House, Harwell Science and Innovation Campus , Didcot , Oxfordshire OX11 0DE , UK

## Abstract

Reversible addition–fragmentation chain transfer (RAFT) dispersion polymerization of benzyl methacrylate (BzMA) is utilized to prepare a series of poly(stearyl methacrylate)–poly(benzyl methacrylate) (PSMA–PBzMA) diblock copolymer nano-objects at 90 °C directly in mineral oil. Polymerization-induced self-assembly (PISA) occurs under these conditions, with the resulting nanoparticles exhibiting spherical, worm-like or vesicular morphologies when using a relatively short PSMA_13_ macromolecular chain transfer agent (macro-CTA), as confirmed by transmission electron microscopy (TEM) and small-angle X-ray scattering (SAXS) studies. Only kinetically-trapped spherical nanoparticles are obtained when using longer macro-CTAs (*e.g.* PSMA_18_ or PSMA_31_), with higher mean degrees of polymerization (DPs) for the PBzMA core-forming block simply producing progressively larger spheres. SAXS is used for the first time to monitor the various morphological transitions that occur *in situ* during the RAFT dispersion polymerization of BzMA when targeting either spheres or vesicles as the final copolymer morphology. This powerful characterization technique enables the evolution of particle diameter, mean aggregation number, number of copolymer chains per unit surface area (*S*_agg_) and the distance between adjacent copolymer chains at the core–shell interface (*d*_int_) to be monitored as a function of monomer conversion for kinetically-trapped spheres. Moreover, the gradual evolution of copolymer morphology during PISA is confirmed unequivocally, with approximate ‘lifetimes’ assigned to the intermediate pure sphere and worm morphologies when targeting PSMA_13_–PBzMA_150_ vesicles. Within vesicle phase space, the membrane thickness (*T*_m_) increases monotonically with PBzMA DP. Furthermore, a combination of dynamic light scattering (DLS), TEM and *post mortem* SAXS studies indicate that the lumen volume is reduced while the overall vesicle dimensions remain essentially constant. Thus the constrained vesicles grow inwards, as recently reported for an aqueous PISA formulation. This suggests a universal vesicle growth mechanism for all PISA formulations.

## Abstract 2

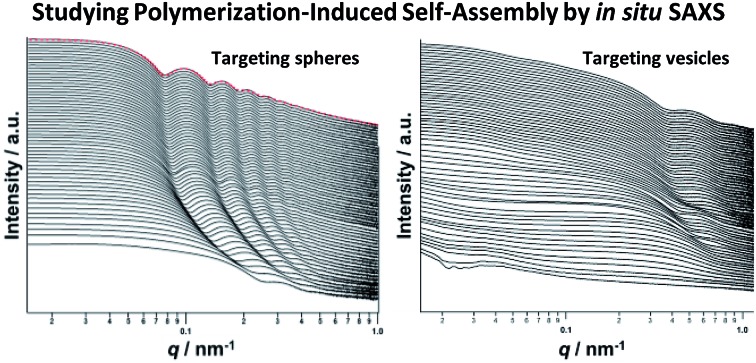
 *In situ* SAXS studies reveal the evolution of copolymer morphology during the PISA synthesis of diblock copolymer nano-objects in mineral oil.

## Introduction

It has been known for more than fifty years that diblock copolymers self-assemble to form well-defined nanoparticles when dispersed in a selective solvent for one of the blocks.^1^–^4^ For example, there is a vast range of literature describing the micellar self-assembly of polystyrene-based block copolymers in non-polar media: spherical morphologies are obtained in most cases,^5^–^11^ but examples of worm-like (or cylindrical)^9^–^13^ and vesicular^11^ morphologies have also been reported. More recently, metal-containing diblock copolymers have been utilized for the formation of cylindrical micelles in *n*-alkanes.^14^–^16^ The commercial potential for diblock copolymer nanoparticles dispersed in non-polar solvents was highlighted by Zheng *et al.*, who reported that spherical nanoparticles of approximately 40 nm diameter offer enhanced boundary lubrication performance when dispersed in base oil.^17^ Moreover, self-assembled block copolymer nanoparticles have been shown to act as effective dispersants for diesel soot, which in turn minimizes wear and hence improves engine efficiency and long-term performance.^18^,^19^

Recently, there has been considerable interest in the development of polymerization-induced self-assembly (PISA), particularly using reversible addition–fragmentation chain transfer (RAFT)^20^–^22^ dispersion polymerization.^23^–^27^ PISA provides an efficient and versatile route to diblock copolymer nanoparticles directly at high solids without the need for post-polymerization processing, making this approach amenable to scale-up.^28^ Most of the PISA literature has focused on optimizing aqueous^26^,^29^–^40^ or alcoholic^41^–^53^ formulations. In contrast, there are relatively few examples of suitable PISA formulations conducted in non-polar solvents such as *n*- or *iso*-alkanes.^54^–^62^ Charleux and co-workers evaluated dithiobenzoate and trithiocarbonate RAFT chain transfer agents (CTAs) for the polymerization of methyl acrylate in *iso*-dodecane.^54^,^55^ However, broad molecular weight distributions and low blocking efficiencies (*i.e.* inefficient re-initiation of the macro-CTA) were achieved, suggesting rather poor control. Fielding *et al.*^57^ reported reasonably well-controlled RAFT polymerizations for the synthesis of poly(lauryl methacrylate)–poly(benzyl methacrylate) (PLMA–PBzMA) diblock copolymer nanoparticles in *n*-heptane *via* PISA. In this case, either spheres, worms or vesicles could be obtained provided that the PLMA stabilizer block was sufficiently short to enable efficient sphere–sphere fusion to occur during PISA. The construction of a phase diagram facilitated reproducible targeting of the worm phase, with these highly anisotropic nanoparticles forming free-standing gels in *n*-heptane at 20 °C.^57^ Derry *et al.*^28^ recently revisited this RAFT dispersion polymerization formulation and developed a highly convenient ‘one-pot’ protocol for the synthesis of PLMA-PBzMA spheres in mineral oil at high solids. This work highlights the potential industrial relevance of such PISA formulations.

Small-angle X-ray scattering (SAXS) techniques have been employed to characterize nanoparticle morphologies obtained by various PISA formulations.^38^,^48^,^58^,^62^–^70^ In particular, thermally-induced micelle-to-unimer^64^,^65^ and worm-to-sphere^58^,^64^,^65^ transitions have been studied, as well as the evolution of vesicle dimensions on increasing the mean degree of polymerization (DP) of the core-forming block.^67^ Most notably for non-polar formulations, heating a free-standing PLMA–PBzMA worm gel in *n*-dodecane to 160 °C resulted in the formation of a free-flowing dispersion of spheres.^58^ This change in copolymer morphology was attributed to ingress of hot solvent leading to surface plasticization of the core-forming PBzMA block, as indicated by variable-temperature ^1^H NMR studies. Such solvation lowers the packing parameter^30^ and hence drives the worm-to-sphere transition, which was confirmed by transmission electron microscopy (TEM) studies.^58^ SAXS was particularly useful for characterizing this specific formulation, since the reduction in the mean worm contour length (*L*_w_) could be monitored on heating from 20 °C (*L*_w_ ≈ 600 nm) to 90 °C (*L*_w_ ≈ 350 nm), with spherical nanoparticles of ∼17 nm diameter being observed at 160 °C. In related work, Lowe and co-workers used TEM and dynamic light scattering (DLS) to demonstrate a worm-to-sphere transition for PSMA–PPPMA nanoparticles in *n*-tetradecane^59^ and *n*-octane,^61^ with ^1^H NMR spectroscopy confirming a similar surface plasticization effect for the core-forming PPPMA block on heating to 95 °C. Recently, SAXS has been utilized to characterize microphase separation within block copolymer microparticles,^71^ with time-resolved studies being conducted during the synthesis of poly(methyl methacrylate)–poly(benzyl methacrylate) (PMMA–PBzMA) block copolymers *via* RAFT dispersion polymerization in supercritical CO_2_.^72^

**Scheme 1:**
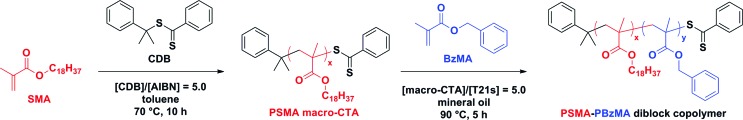
Synthesis of a poly(stearyl methacrylate) (PSMA) macro-CTA *via* RAFT solution polymerization in toluene at 70 °C, followed by RAFT dispersion polymerization of benzyl methacrylate (BzMA) in mineral oil at 90 °C.

Herein we report the PISA synthesis of poly(stearyl methacrylate)–poly(benzyl methacrylate) (PSMA–PBzMA) diblock copolymer nano-objects directly in mineral oil (see Scheme 1). We demonstrate that PSMA offers significant advantages over PLMA in terms of both blocking efficiency and control during the RAFT dispersion polymerization of BzMA. A detailed phase diagram is constructed for this new dispersion polymerization formulation using TEM, while DLS and SAXS are utilized to characterize the nanoparticle dispersions. In particular, we utilize a synchrotron source to conduct SAXS studies of the *in situ* evolution of the copolymer morphology during PISA. SAXS provides remarkably detailed insights regarding the sphere-to-worm and worm-to-vesicle transitions during this non-aqueous PISA formulation and also sheds new light on the mechanism of *in situ* vesicle growth.

## Results and discussion

### Synthesis of macro-CTAs

RAFT solution polymerization of stearyl methacrylate (SMA) was conducted in toluene at 70 °C using cumyl dithiobenzoate (CDB) as a CTA. Three PSMA macro-CTAs were characterized using ^1^H NMR spectroscopy and the mean degree of polymerization (DP) was calculated to be 13, 18 or 31 (see ESI, Table S1†). Each homopolymerization was quenched at 72% to 76% conversion in order to avoid monomer-starved conditions, thus ensuring the retention of RAFT end-groups.^73^,^74^ This is usually required for high blocking efficiencies and hence well-defined PSMA–PBzMA diblock copolymers. Each PSMA macro-CTA had a polydispersity (*M*_w_/*M*_n_) of ≤1.24, which is consistent with previous studies reporting well-controlled RAFT syntheses under these conditions.^57^ A typical kinetic study of the synthesis of a PSMA_31_ macro-CTA *via* RAFT solution polymerization was conducted (Fig. S3a†). After an initial induction period, first-order kinetics were observed prior to quenching at 72% conversion after 10 h. Gel permeation chromatography (GPC) analysis indicated a linear evolution of molecular weight with conversion (Fig. S3b†).

### PSMA_18_–PBzMA_*x*_ and PSMA_31_–PBzMA_*x*_ diblock copolymer spheres

BzMA monomer was polymerized using two of the low polydispersity PSMA macro-CTAs (DP = 18, or 31) in turn *via* RAFT dispersion polymerization (see ESI, Table S2†). In all cases, ≥97% BzMA conversion was achieved within 5 h at 90 °C, as judged by ^1^H NMR spectroscopy. Only spherical morphologies were obtained when using a longer PSMA stabilizer block (DP = 18 or 31). This indicates that the upper limit PSMA DP for access to higher order morphologies (*i.e.* worms or vesicles) is relatively low for this PISA formulation in mineral oil. Longer PSMA stabilizer blocks confer enhanced steric stabilization, which prevents the efficient 1D fusion of multiple spheres and therefore the formation of anisotropic worms. Similar observations were reported for PLMA–PBzMA diblock copolymers prepared *via* RAFT dispersion polymerization of BzMA in *n*-heptane,^57^*n*-dodecane^58^ and mineral oil.^28^ In these earlier studies, the upper limit PLMA stabilizer DP which allowed access to higher order morphologies was 16–18. Given the relative molecular volumes of the LMA (C_12_ side-chain) and SMA (C_18_ side-chain) repeat units, it is reasonable that using PSMA_18_ only allows access to spheres. Clearly, PSMA_13_ has a comparable molecular volume to that of PLMA_18_, which is why using the former macro-CTA allows access to worms and vesicles, as well as spheres.

**Fig. 1:**
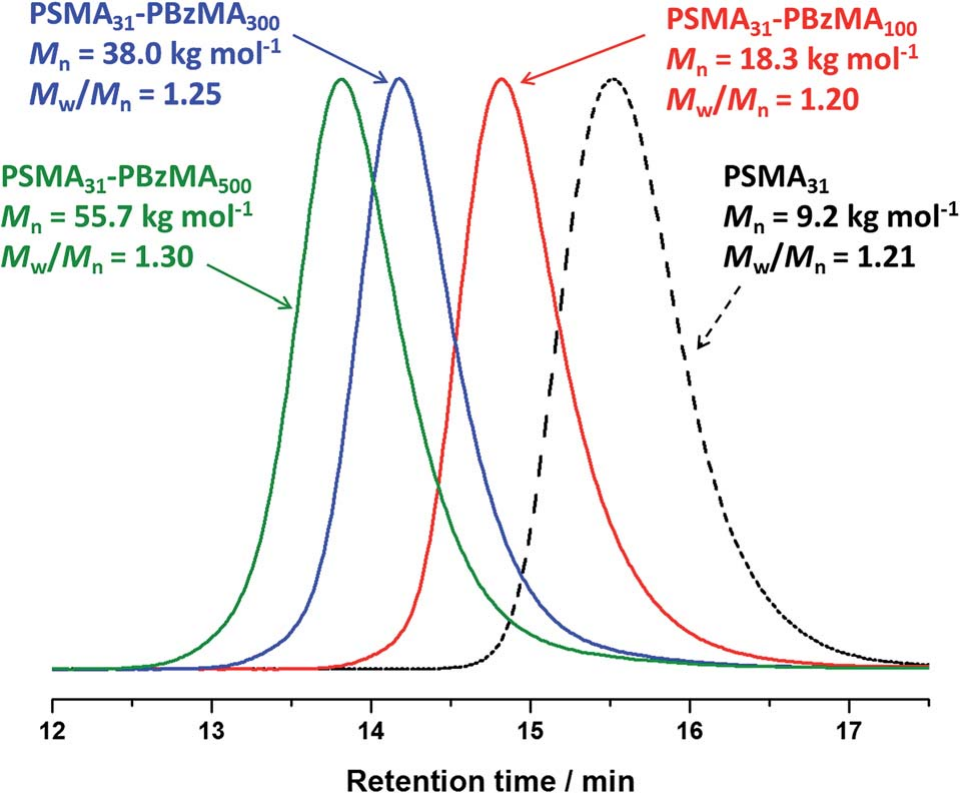
THF gel permeation chromatograms (*vs.* poly(methyl methacrylate) standards) obtained for three PSMA_31_–PBzMA_*x*_ diblock copolymers prepared *via* RAFT dispersion polymerization of BzMA in mineral oil at 90 °C at 20% w/w solids. The precursor PSMA_31_ macro-CTA (prepared in toluene at 70 °C at 40% w/w solids; black dashed curve) is also shown as a reference.

Compared to related RAFT dispersion polymerization syntheses conducted in non-polar media,^54^–^58^ the present PSMA–PBzMA formulation enables relatively narrow molecular weight distributions to be obtained even when targeting PBzMA DPs as high as 500, which corresponds to an experimentally determined *M*_n_ of ∼56 kg mol^–1^ (Fig. 1). GPC analysis of PSMA_31_–PBzMA_*x*_ (*x* ≤ 500) diblock copolymers in THF eluent indicates *M*_w_/*M*_n_ values ranging between 1.19 and 1.30, which suggests good RAFT control. Also, the unimodal nature of these curves and the clear shift from the original PSMA_31_ macro-CTA indicates relatively high blocking efficiencies. In contrast, relatively broad molecular weight distributions (*M*_w_/*M*_n_ > 1.50) were reported by Fielding *et al.* when targeting *x* values above 300 for PLMA_37_–PBzMA_*x*_ diblock copolymers *via* closely-related PISA syntheses conducted in *n*-heptane.^57^ However, it is not yet understood why simply using a PSMA macro-CTA instead of a PLMA macro-CTA leads to significantly better pseudo-living character during the dispersion polymerization of BzMA.

**Fig. 2:**
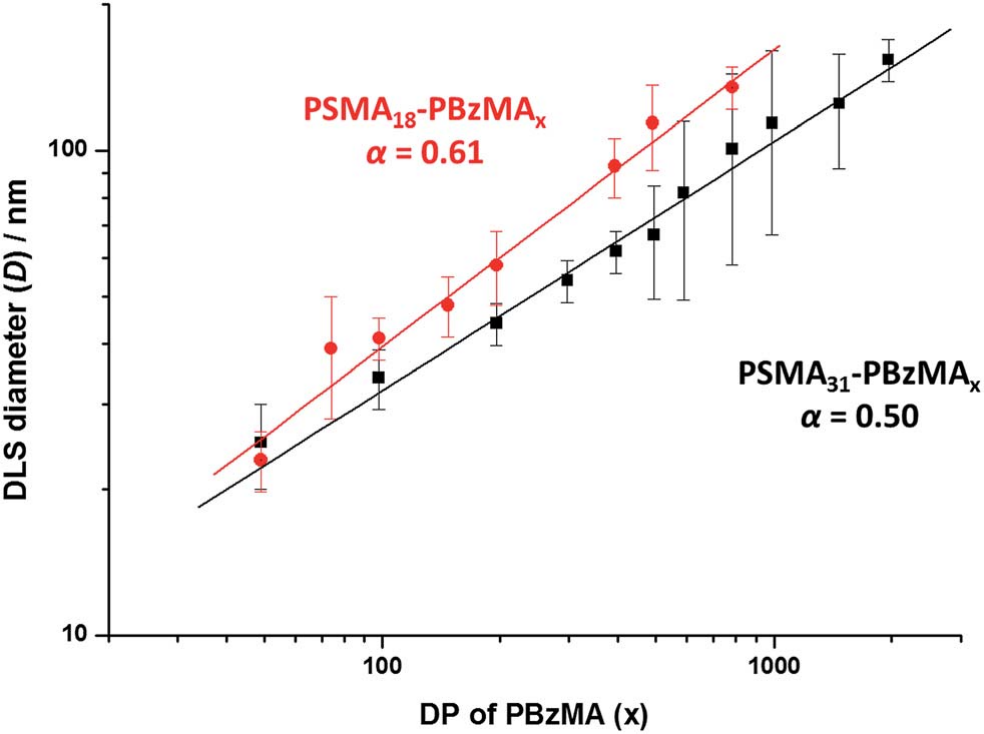
Relationship between intensity-average sphere diameter (*D*) and target DP of the PBzMA block (*x*) for series of PSMA_18_–PBzMA_*x*_ (red circles) and PSMA_31_–PBzMA_*x*_ (black squares) diblock copolymer spheres prepared *via* RAFT dispersion polymerization of BzMA in mineral oil at 90 °C. The error bars represent the standard deviation of the diameter and *α* is the scaling factor.

A series of spherical nanoparticles with tunable diameters was conveniently prepared in mineral oil at 20% w/w solids simply by varying the target DP of the core-forming PBzMA block when using a PSMA macro-CTA with a sufficiently high DP. For example, PSMA_18_–PBzMA_*x*_ spheres ranging from 23 to 135 nm diameter (as judged by DLS) were obtained when targeting *x* values of 50 to 800. Similarly, well-defined PSMA_31_–PBzMA_*x*_ spheres of 25 to 154 nm diameter were produced for *x* = 50 to 2000. The mean sphere diameter, *D*, is related to the mean DP of the core-forming block, *x*, by a scaling exponent, *α*, as indicated by the equation *D* ∼ *kx*^*α*^ where *k* is a constant.^75^,^76^ Fig. 2 shows double-logarithmic plots of *D*, as judged by DLS, against *x* for each series of PSMA_18_–PBzMA_*x*_ and PSMA_31_–PBzMA_*x*_ spheres. A clear relationship is observed in each case, which enables the corresponding scaling exponent (*α*) to be determined. This parameter provides important information regarding the behavior of the PBzMA core-forming chains. For the PSMA_31_–PBzMA_*x*_ series we find that *α* = 0.50, which corresponds to unperturbed PBzMA chains.^75^,^76^,^80^ According to the literature, such low *α* values suggest weak segregation (and minimal solvation).^75^,^76^,^80^ On the other hand, we find that *α* = 0.61 for the PSMA_18_–PBzMA_*x*_ series, indicating that the PBzMA chains are more stretched and may have a finite degree of solvation. This means that, for a given PBzMA_*x*_ block (where *x* > 50), larger spheres are always obtained when using the shorter PSMA_18_ stabilizer block. For example, DLS studies indicate that spheres obtained when targeting a core-forming PBzMA DP of 400 are larger when using the PSMA_18_ macro-CTA (93 nm) compared to the PSMA_31_ macro-CTA (62 nm).

### *In situ* SAXS studies of the PISA synthesis of PSMA_31_–PBzMA_2000_ spheres

**Fig. 3:**
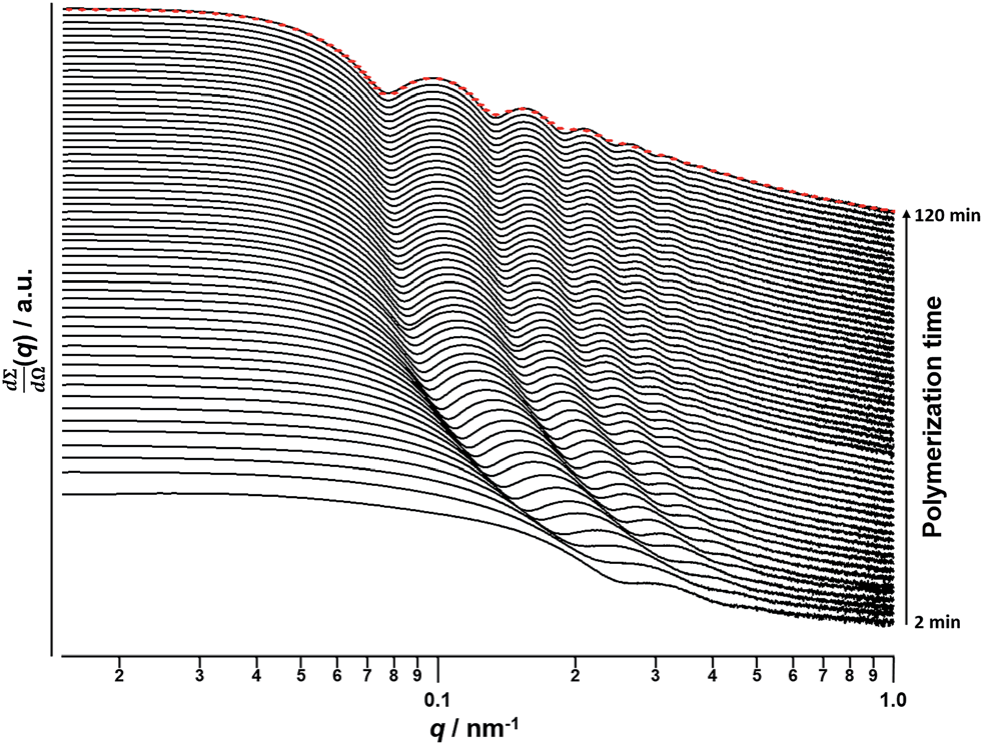
SAXS patterns obtained *in situ* during the PISA synthesis of PSMA_31_–PBzMA_2000_ diblock copolymer spheres at 90 °C in mineral oil at 10% w/w solids. Red dashes indicate the data fit to the final SAXS pattern recorded after 120 min using a spherical micelle model.^77^–^79^

A synchrotron X-ray source was used to acquire SAXS patterns *in situ* during the PISA synthesis of PSMA_31_–PBzMA_2000_ diblock copolymer spheres at 90 °C in mineral oil at 10% w/w solids. The sample cell was a 2 mm glass capillary and scattering patterns were recorded every 2 min for 120 min (Fig. 3). The onset of micellization occurs when the growing PBzMA chains become sufficiently long to induce nucleation.^26^,^27^ This occurred within around 2 min of the polymerization, as indicated by the presence of a local minimum at *q* ∼ 0.23 nm^–1^ (where *q* = 4π sin *θ*/*λ* is the length of the scattering vector, *λ* is the wavelength of X-ray radiation and *θ* is one-half of the scattering angle). The characteristic length scale corresponding to this feature is the mean core radius of the spherical diblock copolymer nanoparticles (*R*_s_), which was observed to be 15 nm. Since the PISA synthesis was conducted at 10% w/w solids, it was necessary to incorporate an appropriate structure factor^81^ into a well-known spherical micelle model^77^–^79^ in order to obtain satisfactory fits to the SAXS patterns.

**Fig. 4:**
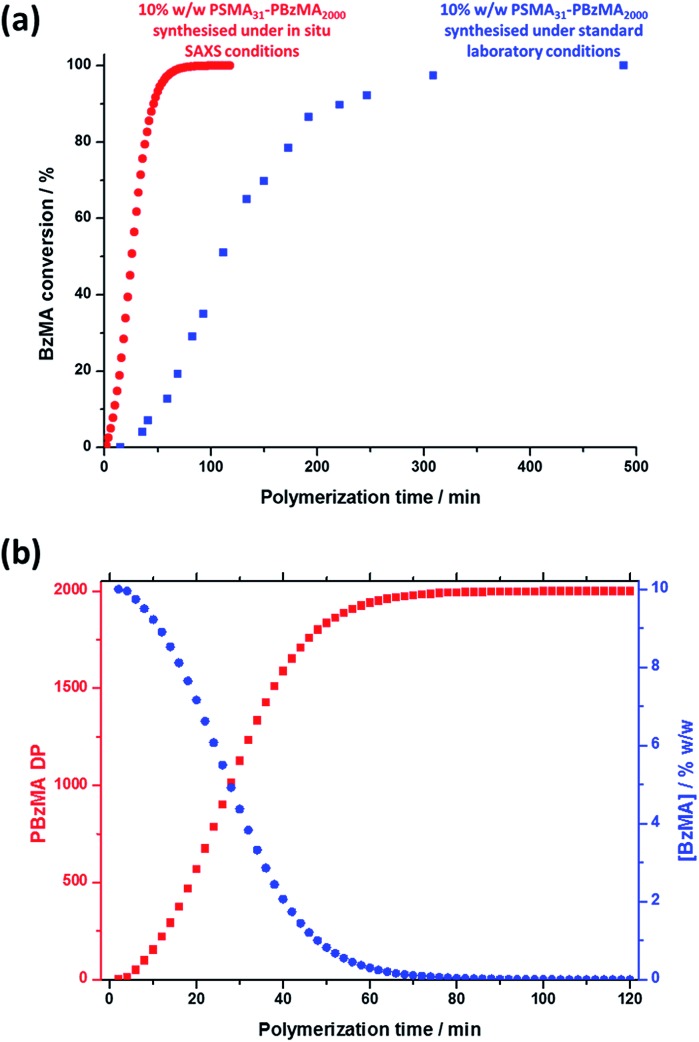
(a) Conversion *vs.* time curve (blue squares) for the RAFT dispersion polymerization of BzMA in mineral oil at 90 °C when targeting PSMA_31_–PBzMA_2000_ block copolymer spheres at 10% w/w solids using T21s initiator under normal laboratory conditions and the renormalized conversion *vs.* time curve (red circles) calculated for the same PISA synthesis during *in situ* SAXS studies. (b) Change in the PBzMA DP (red data) and the concentration of BzMA monomer ([BzMA], blue data) during the *in situ* SAXS studies when targeting PSMA_31_–PBzMA_2000_ spheres.

Monitoring this minimum as it shifts to lower *q* (larger radii) as the BzMA polymerization proceeded provides useful information regarding the kinetics of nanoparticle growth. However, in order to fit the SAXS data shown in Fig. 3 to a spherical micelle model,^77^–^79^ the *instantaneous BzMA conversion* is required, since this in turn determines the mean DP and hence the molecular volume occupied by a single growing core-forming (PBzMA) block within the sphere is given by *V*_s_ = (DP_PBzMA_*M*_n,BzMA_)/(*N*_A_*ρ*), where *M*_n,BzMA_ corresponds to the molecular weight of the one BzMA unit within the PBzMA block and *ρ* is the density of PBzMA. No further change in the SAXS patterns shown in Fig. 3 was taken to signify the end of the polymerization. Unfortunately, the BzMA polymerization was complete within 120 min during the *in situ* SAXS studies, whereas around 500 min was required for the same formulation in a typical laboratory-scale synthesis (∼20 mL reaction volume) conducted using an oil bath and stirrer hot plate. A possible reason for this significant increase in polymerization rate could be additional radical species generated by the intense X-ray photon flux provided by the synchrotron source.^82^,^83^ The ∼125 μL reaction volume of the capillary used for the SAXS studies precludes sampling of the polymerizing reaction mixture. Instead, intermediate BzMA conversions were calculated by renormalizing the kinetic data set obtained for the laboratory-scale synthesis. More specifically, a sigmoid function was used to calculate intermediate BzMA conversions (see Fig. 4a and ESI†) since this best described the conversion *vs.* time curve.^84^ The resulting BzMA conversions were subsequently used to calculate the instantaneous PBzMA DP during the PISA synthesis (see Fig. 4b, red data). It must be noted that due to the nature of the renormalization using the sigmoid function, the predicted kinetic data for the *in situ* SAXS measurements are a smooth representation of the somewhat scattered experimental data obtained under standard laboratory conditions.

**Fig. 5:**
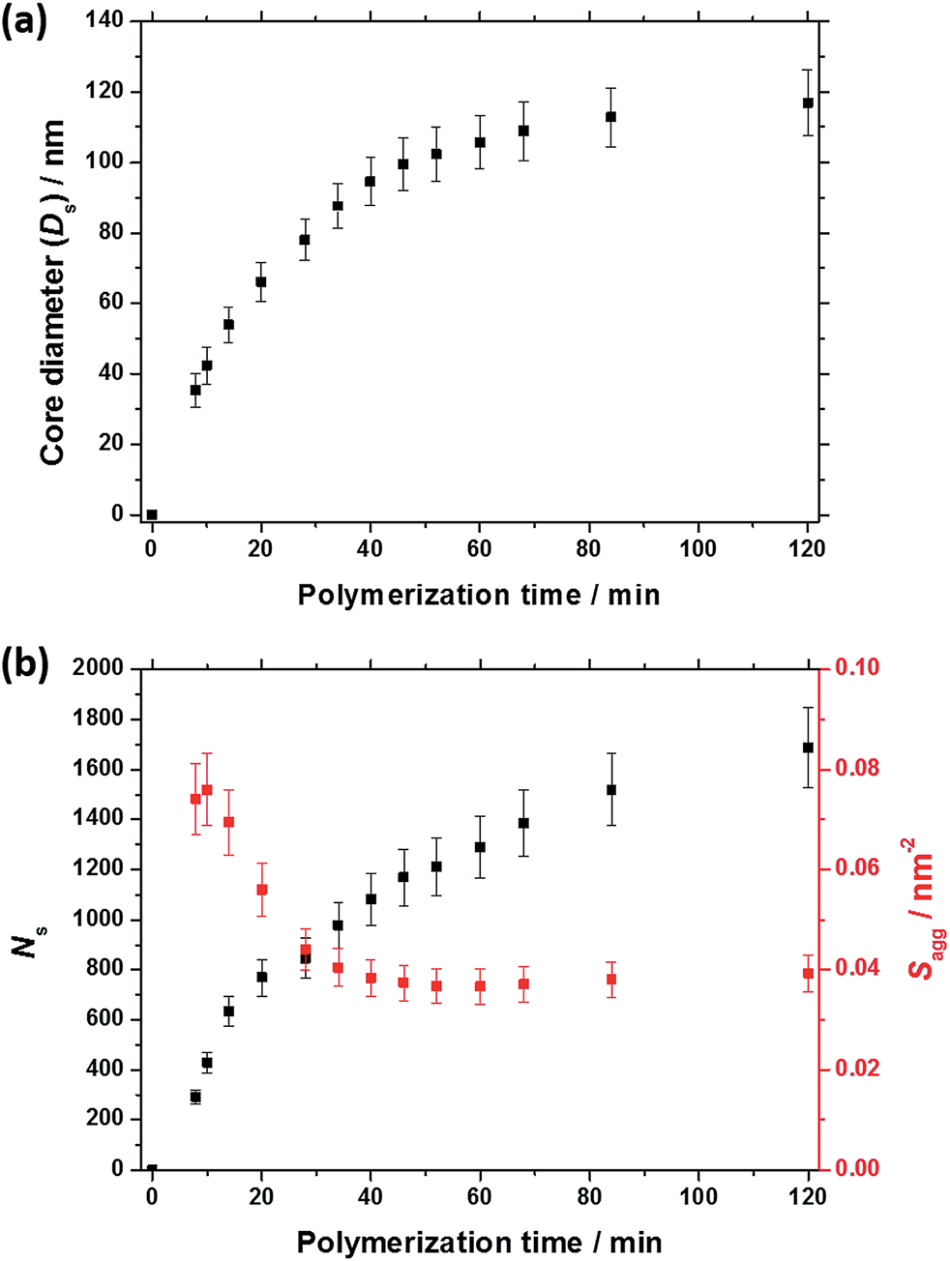
(a) Evolution of the mean core diameter (*D*_co_) and (b) mean aggregation number (*N*_agg_, black data set) and number of copolymer chains per unit surface area (*S*_agg_, red data set) during the PISA synthesis of PSMA_31_–PBzMA_2000_ diblock copolymer spheres, as judged by *in situ* SAXS studies.

As expected, the spherical core diameter of the growing nanoparticles (*D*_s_) increases monotonically with polymerization time (see Fig. 5a and Table 1). At the end of the BzMA polymerization, at least six minima are visible in the final scattering pattern (120 min, Fig. 3), indicating a relatively narrow size distribution for the resulting PSMA_31_–PBzMA_2000_ spheres. Data fitting for various SAXS patterns during the RAFT dispersion polymerization of PSMA_31_–PBzMA_2000_ spheres indicated essentially no solvent associated with the core-forming PBzMA block, which is consistent with the PSMA_31_–PBzMA_*x*_ data set shown in Fig. 2. Moreover, ^1^H NMR studies of the latter laboratory-scale syntheses (data not shown) confirmed that the volume fraction of solvent within the core domain (*x*_sol_) is essentially zero. Thus, when fitting SAXS patterns recorded during the polymerization, the mean number of copolymer chains per sphere (*N*_s_) was calculated based solely on the volume fraction of BzMA monomer within the core domain (*φ*_BzMA_), *R*_s_ and *V*_s_ as shown below.1
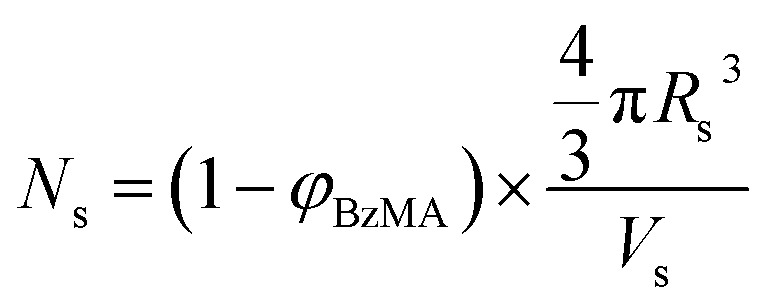



**Table 1:** Evolution of BzMA conversion, the mean degree of polymerization (DP) of the core-forming PBzMA block, the molecular volume occupied by a single PBzMA chain within the spherical core (*V*_s_), the spherical core diameter (*D*_s_ = 2*R*_s_), volume fraction of BzMA monomer within the core domain (*φ*_BzMA_), mean aggregation number of a sphere (*N*_s_), number of copolymer chains per unit surface area (*S*_agg_) and mean distance between adjacent chains at the core–shell interface (*d*_int_) during the PISA synthesis of PSMA_31_–PBzMA_2000_ diblock copolymer spheres. The standard deviation in *D*_s_ (*σ*_*D*_s__ = 2*σ*_*R*_s__) and the associated error in *V*_s_, *N*_s_, *S*_agg_ and *d*_int_ are indicated

Time/min	BzMA conversion/%	PBzMA DP	*V*_s_/nm^3^	*D*_s_/nm	*φ*_BzMA_	*N*_s_	*S*_agg_/nm^–2^	*d*_int_/nm
8	5.0	99	25 ± 2	35 ± 5	0.691	292 ± 28	0.074 ± 0.007	3.7 ± 0.4
10	7.8	155	39 ± 4	42 ± 5	0.586	429 ± 41	0.076 ± 0.007	3.6 ± 0.3
14	14.7	294	73 ± 7	54 ± 5	0.437	633 ± 60	0.069 ± 0.007	3.8 ± 0.4
20	28.4	569	141 ± 13	66 ± 6	0.282	769 ± 73	0.056 ± 0.005	4.2 ± 0.4
28	50.8	1015	252 ± 24	78 ± 6	0.147	845 ± 80	0.044 ± 0.004	4.8 ± 0.5
34	66.8	1335	331 ± 32	88 ± 6	0.083	978 ± 93	0.040 ± 0.004	5.0 ± 0.5
40	79.3	1587	394 ± 37	95 ± 7	0.042	1082 ± 103	0.038 ± 0.004	5.1 ± 0.5
46	87.9	1758	436 ± 41	100 ± 8	0.018	1168 ± 111	0.037 ± 0.004	5.2 ± 0.5
52	93.2	1864	463 ± 44	102 ± 8	0.004	1210 ± 115	0.037 ± 0.003	5.2 ± 0.5
60	97.0	1939	481 ± 46	106 ± 8	0.000	1289 ± 122	0.037 ± 0.003	5.2 ± 0.5
68	98.7	1973	490 ± 47	109 ± 8	0.000	1385 ± 132	0.037 ± 0.004	5.2 ± 0.5
84	99.8	1995	495 ± 47	113 ± 8	0.000	1519 ± 144	0.038 ± 0.004	5.1 ± 0.5
120	100	2000	496 ± 47	117 ± 9	0.000	1688 ± 160	0.039 ± 0.004	5.0 ± 0.5

Values for *φ*_BzMA_ were estimated *via* centrifugation of selected dispersions of PSMA_31_–PBzMA_*x*_ spheres (obtained at full BzMA conversion *via* laboratory-scale syntheses) to which varying amounts of BzMA monomer and additional mineral oil had been added in order to replicate specific intermediate BzMA conversions during the synthesis of PSMA_31_–PBzMA_2000_ spheres in the *in situ* SAXS studies. Firstly, the BzMA–swollen PSMA_31_–PBzMA_*x*_ spheres were heated at 90 °C for 1 h and then centrifuged at 13 000 rpm for 1 to 10 h at 20 °C to ensure complete sedimentation of the spheres. Since centrifugation was not possible at 90 °C, it is assumed that the amount of BzMA monomer within the PBzMA cores is the same at 20 °C and 90 °C. Each supernatant was then analyzed for its BzMA content against an internal standard (triethoxymethylsilane) *via*^1^H NMR spectroscopy (see ESI† for further details). The experimentally-determined values of *φ*_BzMA_ at particular BzMA conversions were then fitted to a logarithmic decay function (*R*^2^ > 0.95), which was subsequently utilized to calculate *φ*_BzMA_ values for all entries in Table 1 *via* interpolation. Eqn (1) was then used to calculate the corresponding *N*_s_ values. According to the SAXS fittings, the uncertainty in *R*_s_ is small, hence the error in *N*_s_ is dominated by that associated with *V*_s_, which is in turn dictated by the molecular weight distribution (MWD) of the growing core-forming PBzMA block. Given that the PSMA_31_ stabilizer block is relatively short, this MWD is approximately the same as that of the diblock copolymer. However, since the *in situ* SAXS experiments were conducted on such a small scale, it was not feasible to determine the copolymer MWD at intermediate times during the polymerization. Therefore, the maximum error in *V*_s_ at any given time during the polymerization was estimated from the final MWD obtained for the laboratory-scale synthesis of the equivalent PSMA_31_–PBzMA_2000_ spheres. The unimodal MWD determined by THF GPC was fitted to a Gaussian model to determine its standard deviation (see ESI†), which was found to be approximately 9.5%. Since the PISA synthesis conducted under *in situ* SAXS conditions proceeded much faster than standard laboratory conditions, it is possible that a broader MWD is observed for the copolymers synthesized in the former case. However, several recent reports of PISA syntheses conducted in non-polar solvents indicate that there is no correlation between copolymer MWD and the final copolymer morphology – even highly polydisperse copolymer chains (*M*_w_/*M*_n_ > 2.0) can self-assemble to give well-defined nano-objects.^85^,^86^

*N*_s_ gradually increased with polymerization time, as indicated in Fig. 5b (black data). This is not unexpected in view of recent observations made by both Jones *et al.*^70^ and Zhang and co-workers^87^ for non-aqueous PISA formulations. Nevertheless, it provides the first direct experimental evidence that the mean number of copolymer chains per nanoparticle increases during PISA syntheses. Likely mechanisms are either efficient fusion between monomer-swollen spheres and/or continuous aggregation of molecularly-dissolved copolymer chains.^70^ The latter seems more likely to occur during the early stages of the polymerization (just after nucleation), rather than in the latter stages. The average number of copolymer chains per unit surface area (*S*_agg_) during the polymerization was calculated using eqn (2) below.2
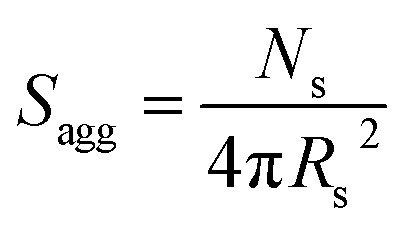



Interestingly, *S*_agg_ values (Fig. 5b, red data) decrease from 0.075 nm^–2^ to a limiting value of approximately 0.04 nm^–2^ after around 40 min, suggesting an optimum surface packing density of copolymer chains within the sterically-stabilized PSMA_31_–PBzMA_2000_ spherical nanoparticles.^70^

The average distance between adjacent chains at the core–shell interface (*d*_int_) was calculated using eqn (3) below.^76^3
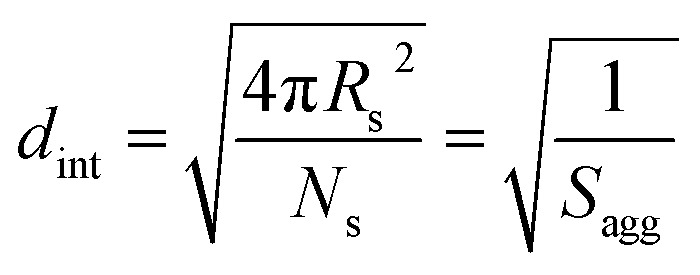



For small spheres (*i.e.*, *D*_s_ = 35.4 nm), *d*_int_ was calculated to be 3.67 nm after 8 min (or 5.0% BzMA conversion, which corresponds to PSMA_31_–PBzMA_99_). This is comparable to that reported by Förster *et al.*^76^ for similar-sized polystyrene–poly(4-vinyl pyridine) block copolymer micelles, for which *d*_int_ was found to be 3.20 nm. Subsequently, *d*_int_ increased up to 5.04 nm at full conversion (*i.e.*, PSMA_31_–PBzMA_2000_; *D*_s_ = 116.9 nm), indicating that copolymer chains with longer core-forming PBzMA blocks occupy a larger surface area at the core–shell interface.

### PSMA_13_–PBzMA_*x*_ block copolymer syntheses and corresponding phase diagram

**Fig. 6:**
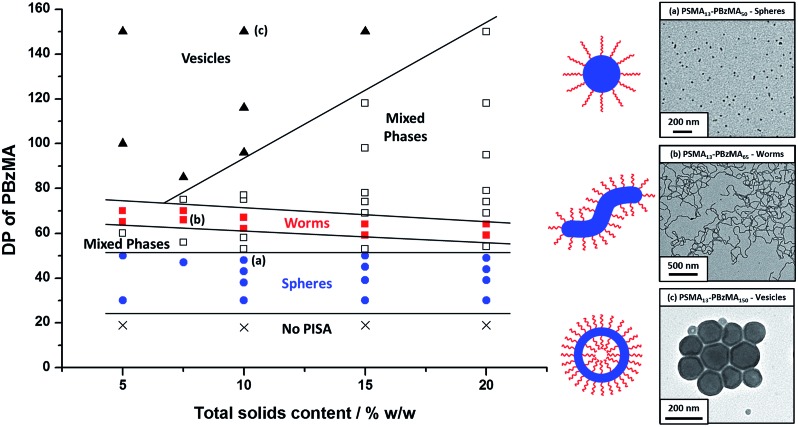
Phase diagram constructed for PSMA_13_–PBzMA_*x*_ diblock copolymer nanoparticles prepared by RAFT dispersion polymerization of BzMA in mineral oil using a PSMA_13_ macro-CTA and T21s initiator at 90 °C ([PSMA_13_]/[T21s] molar ratio = 5.0). The *post mortem* diblock copolymer morphologies obtained at full conversion were assigned on the basis of TEM studies. TEM images (a), (b) and (c) correspond to typical examples of the three pure copolymer morphologies (spheres, worms and vesicles) respectively.

Utilizing a shorter PSMA_13_ macro-CTA to target PBzMA core-forming block DPs of 20 to 150 at various copolymer concentrations enabled access to spherical, worm-like and vesicular morphologies *at relatively low copolymer concentrations* (≥5% w/w solids). In contrast, well-defined vesicular morphologies were only obtained at copolymer concentrations of at least 12.5% w/w solids for the PISA synthesis of PLMA–PBzMA diblock copolymer nanoparticles, whereas somewhat higher copolymer concentrations (≥17.5% w/w solids) were required to access a pure worm phase.^28^,^57^,^58^ A detailed phase diagram was constructed for the present PSMA_13_–PBzMA_*x*_ formulation, with diblock copolymer morphologies assigned *via post mortem* TEM studies (see Fig. 6). Such phase diagrams are essential to ensure reproducible targeting of the desired copolymer morphology. Thus spheres were obtained at all copolymer concentrations investigated (5–20% w/w) when targeting PBzMA block DPs of 30 to 50. As reported for related PISA formulations,^26^,^28^,^34^,^45^,^46^,^57^,^58^,^62^ the worm phase space is relatively narrow and is bounded by mixed phase regions. As expected, pure vesicles were obtained by targeting asymmetric PSMA_13_–PBzMA_*x*_ diblock copolymers (*i.e. x* > 80). However, for PBzMA DPs of up to 150 this phase appears to be confined to copolymer concentrations of 5–15% w/w solids, with mixed phases being obtained at 20% w/w solids. It is perhaps worth emphasizing that the ability to prepare vesicles at copolymer concentrations as low as 5% w/w solids is an important advantage for *in situ* SAXS studies (see later). This is because lower copolymer concentrations minimize structural effects arising from inter-particle interactions.

**Fig. 7:**
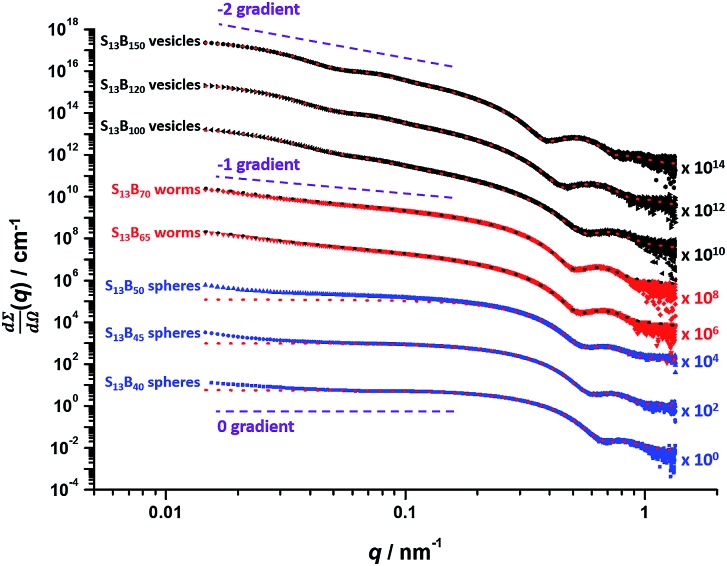
*Post mortem* SAXS patterns and data fits (dashed lines) for 1.0% w/w dispersions of PSMA_13_–PBzMA_*x*_ (denoted as S_13_–B_*x*_ for brevity) diblock copolymer nanoparticles synthesized *via* RAFT dispersion polymerization of BzMA at 10% w/w solids in mineral oil. Purple dashed lines indicate zero, –1 and –2 gradients for guidance.

*Post mortem* SAXS patterns recorded for 1.0% w/w dispersions of eight PSMA_13_–PBzMA_*x*_ diblock copolymer nano-objects (originally prepared at 10% w/w solids; see phase diagram in Fig. 6) are depicted in Fig. 7. Each of the three examples of spherical nanoparticles exhibit an approximate zero gradient at low *q*, as expected.^88^ Some deviations from zero gradient observed at low *q* values could be associated with an aggregation of the spherical micelles. The local minimum observed for each scattering curve at *q* ≈ 0.5–0.7 nm^–1^ gradually shifted to lower *q* on increasing the mean PBzMA DP from 40 to 50, indicating a progressive increase in the sphere dimensions. This is consistent with previously reported PISA syntheses conducted using a fixed stabilizer block DP, where increasing the core-forming block DP led to larger spherical nanoparticles.^33^,^57^ According to theory, rigid rods should exhibit a limiting gradient of –1 at low *q*.^88^ However, TEM studies (see Fig. 6b) suggest that these particular worms exhibit appreciable flexibility. Nevertheless, the SAXS patterns recorded for PSMA_13_–PBzMA_65_ and PSMA_13_–PBzMA_70_ worms in Fig. 7 do indeed exhibit gradients of approximately –1 at low *q*. For these two copolymer dispersions, the local minimum observed at *q* ≈ 0.5–0.6 nm^–1^ is associated with the mean worm width. Vesicular morphologies were also confirmed for PSMA_13_–PBzMA_100–150_, since SAXS patterns indicated a slope of approximately –2 at low *q* for these three dispersions. For such hollow spheres, there are two characteristic local minima. Firstly, the minimum observed at *q* ≈ 0.4–0.6 nm^–1^ is associated with the vesicle membrane thickness (*T*_m_), which increases monotonically as higher PBzMA DPs are targeted. Secondly, the local minimum observed at *q* ≈ 0.04–0.05 nm^–1^ is characteristic of the overall vesicle dimensions. Interestingly, this parameter remains relatively constant (109 ± 5 nm) for the series of three PSMA_13_–PBzMA_100–150_ vesicles prepared at 10% w/w solids shown in Fig. 6.

### *In situ* SAXS studies of the PISA synthesis of PSMA_13_–PBzMA_150_ vesicles

**Fig. 8:**
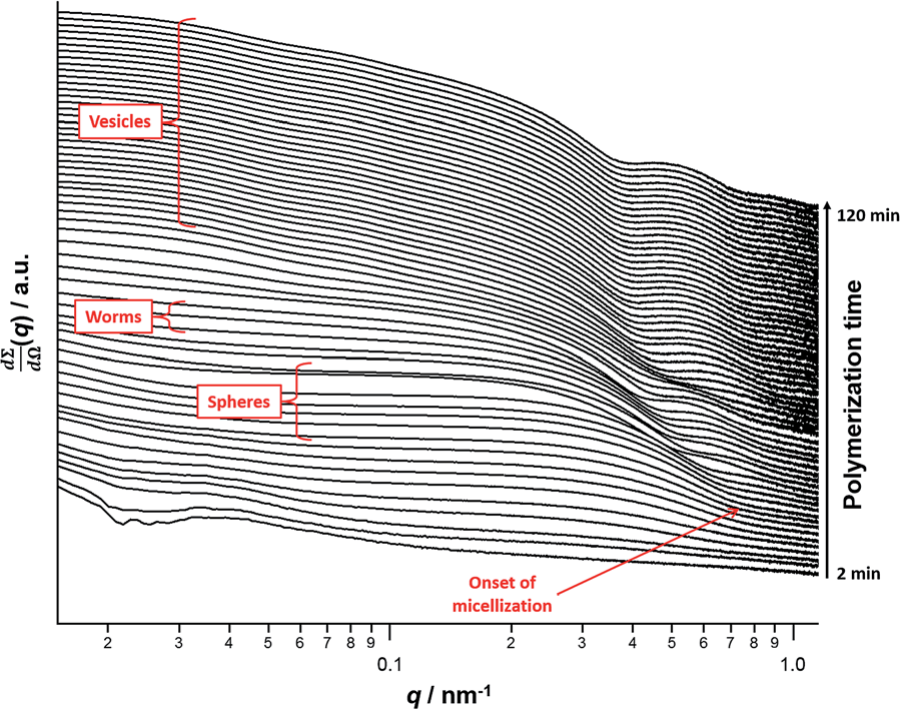
*In situ* SAXS patterns recorded for the PISA synthesis of PSMA_13_–PBzMA_150_ diblock copolymer vesicles prepared at 90 °C in mineral oil at 10% w/w solids. The onset of micellar nucleation is indicated by the red arrow.

A series of *in situ* SAXS patterns were also recorded when targeting PSMA_13_–PBzMA_150_ vesicles at 10% w/w solids in mineral oil. This core-forming block DP was chosen to guarantee access vesicle space (see Fig. 6) while maximizing the time scales for the existence of the intermediate sphere and worm phases. Inspecting Fig. 8, this strategy was clearly successful since the full range of copolymer morphologies is observed, from initially soluble copolymer chains through to the final vesicular morphology *via* intermediate spherical and worm-like nanoparticles.^35^ Again, the polymerization kinetics required renormalization prior to detailed data analyses (see Fig. S4†). In this case, a significantly longer polymerization time (and hence a somewhat higher BzMA conversion) is required for the onset of micellization. Inspecting Fig. 6, it is clear that the critical DP for the core-forming PBzMA block required to induce nucleation is around 30. This is because PSMA_13_–PBzMA_20_ diblock copolymers do not self-assemble in mineral oil at 90 °C, whereas PSMA_13_–PBzMA_30_ diblock copolymer spheres are observed under these conditions. Thus approximately 20% BzMA conversion is required to trigger *in situ* self-assembly for this particular PSMA_13_–PBzMA_150_ PISA formulation. In contrast, when targeting PSMA_31_–PBzMA_2000_ diblock copolymer spheres (Fig. 3), a BzMA conversion of only ∼1.5% is required to achieve the same critical PBzMA DP for micellar nucleation. Spherical nanoparticles are formed just after the onset of micellization, as confirmed by the approximately zero gradient at low *q*.^88^ However, just 10 min after nucleation this low *q* gradient tends towards –1, indicating that the nascent spherical nanoparticles undergo multiple 1D fusion events leading to the formation of highly anisotropic worms. This second morphology is relatively short-lived (∼6 min), which is consistent with the narrow worm phase space observed in the phase diagram (Fig. 6). A mixed phase of worms and vesicles is apparent from 46 to 56 min. This corresponds to a PBzMA DP of 76 to 104 and is consistent with the mixed phase region observed in Fig. 6. Informed by these *in situ* studies, multiple aliquots were taken from the same polymerization conducted under standard laboratory conditions, with particular attention being paid to the above DP interval. TEM analyses confirmed that vesicles are formed from worms *via* octopi (see Fig. S7b†) and jellyfish (see Fig. S5c and S5d†) intermediates. Such transient structures were also reported by Blanazs and co-workers^35^ for an aqueous PISA formulation when targeting vesicles as the final copolymer morphology. This provides the first experimental evidence of octopi and jellyfish intermediates for a non-polar PISA formulation and suggests that the worm-to-vesicle morphology transition *via* such structures is likely to be universal for all vesicles prepared *via* PISA syntheses. Finally, well-defined vesicles are present as a pure phase in the latter stages of the polymerization (∼58–120 min), as indicated by the slope of –2 at low *q*.

**Fig. 9:**
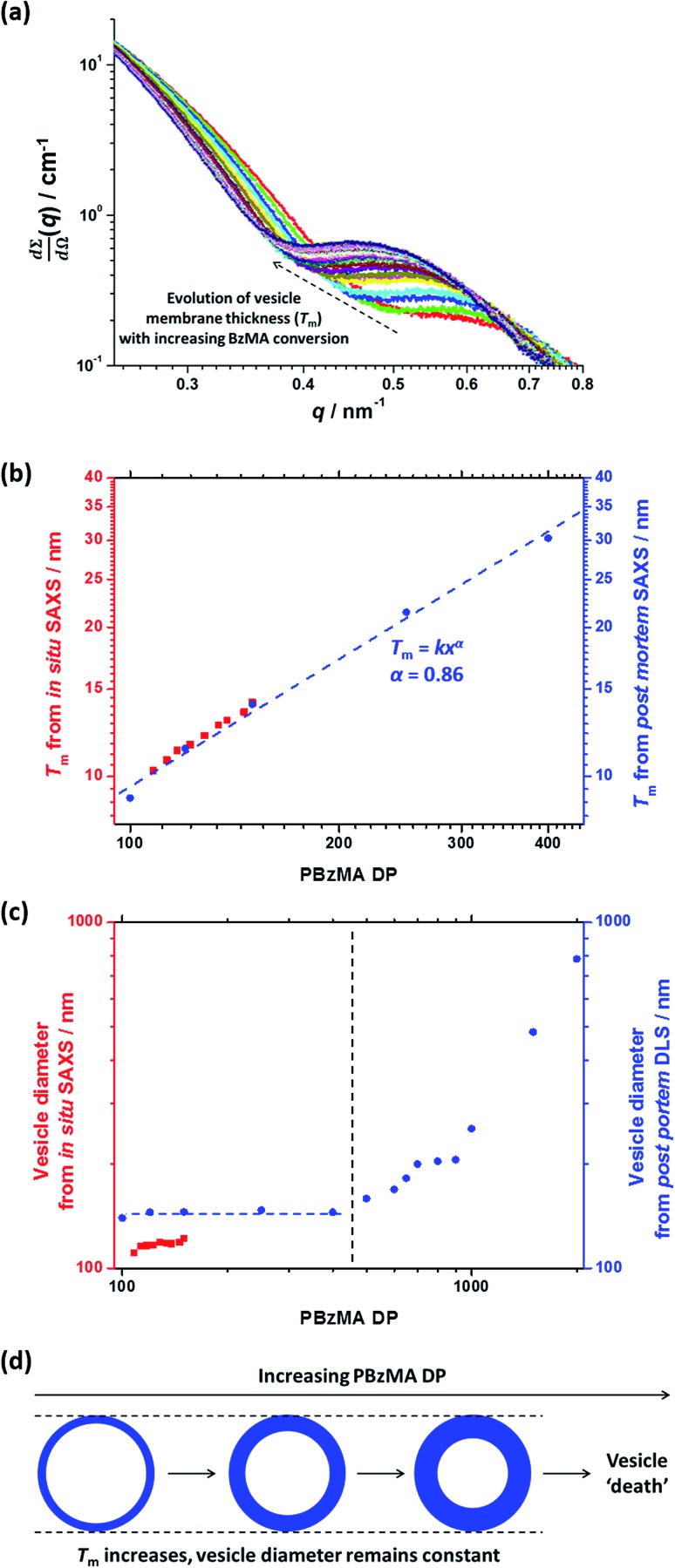
(a) *In situ* SAXS patterns showing the evolution of the vesicular membrane thickness (*T*_m_). (b) Relationship between *T*_m_ and PBzMA DP as judged by *in situ* SAXS when targeting PSMA_13_–PBzMA_150_ vesicles (red squares) and *post mortem* SAXS studies of PSMA_13_–PBzMA_*x*_ vesicles (blue circles). (c) Vesicle diameter as judged by *in situ* SAXS when targeting PSMA_13_–PBzMA_*x*_ vesicles (red squares) and *post mortem* DLS studies of PSMA_13_–PBzMA_*x*_ vesicles (blue data). (d) *T*_m_ increases monotonically when targeting higher PBzMA DPs while the overall vesicle diameter remains relatively constant, thus the lumen volume is gradually reduced during inward vesicle growth until vesicle ‘death’ (or break-up) occurs.

For this particular *in situ* SAXS study (see Fig. 8), the experimental protocol used to renormalize the polymerization kinetics can be validated by comparing the PBzMA DP ranges within which pure spheres, worms and vesicles are observed to those indicated within the phase diagram shown in Fig. 6 (see Table 2). The generally good agreement between the upper and lower DPs at which each pure morphology is observed provides strong evidence that the analytical approach employed to renormalize the kinetic data is indeed valid. It is also worth emphasizing that the relatively well-defined phase boundaries shown in Fig. 6 enable a particularly robust comparison. SAXS patterns assigned to pure vesicles exhibit two local minima: one is a rather subtle feature at *q* ≈ 0.04–0.07 nm^–1^ representing the overall vesicle dimensions and the other is a more pronounced feature at *q* ≈ 0.3–0.7 nm^–1^ that is associated with the vesicle membrane thickness (*T*_m_).^67^Fig. 9a shows selected SAXS patterns taken from Fig. 8 over a much narrower *q* range in order to better illustrate the evolution in *T*_m_ at *q* ≈ 0.3–0.7 nm^–1^. A pure vesicle phase is observed after 58 min, with subsequent data fits indicating that *T*_m_ increases monotonically from 10.3 nm to 14.1 nm for PBzMA DPs ranging from 108 to 150 (see Fig. 9b, red data and Table 3). There is also an apparent increase in the outer core radius (*R*_out_) with increasing PBzMA DP (see Table 3) but this rather modest difference appears to be within the relatively large error associated with these data.

**Table 2:** Comparison of the lower and upper limit PBzMA DPs for the three pure copolymer morphologies (spheres, worms and vesicles) determined by (i) inspecting the phase diagram constructed for PSMA_13_–PBzMA_*x*_ diblock copolymer nanoparticles prepared at 10% w/w solids (Fig. 6) and (ii) *in situ* SAXS analysis of the synthesis of PSMA_13_–PBzMA_150_ vesicles (Fig. 8)

Pure copolymer morphology		PBzMA DP indicated by phase diagram	PBzMA DP indicated by *in situ* SAXS studies
Spheres	Lower limit boundary	25 ± 5	29 ± 3
Spheres	Upper limit boundary	51 ± 1	48 ± 4
Worms	Lower limit boundary	60 ± 1	59 ± 5
Worms	Upper limit boundary	70 ± 1	70 ± 6
Vesicles	Lower limit boundary	93 ± 2	108 ± 4

**Table 3:** Evolution of the BzMA conversion, mean degree of polymerization (DP) for the core-forming PBzMA block, molecular volume of a single PBzMA core-forming block within the membrane (*V*_m_), outer core radius (*R*_out_), membrane thickness (*T*_m_) and inner core radius (*R*_in_ = *R*_out_ – *T*_m_) during the PISA synthesis of PSMA_13_–PBzMA_150_ diblock copolymer vesicles. The associated error in *V*_m_ is indicated and the standard deviation is shown where relevant (±*σ*_*R*_out__, *σ*_*T*_m__, *σ*_*R*_in__)

Time/min	BzMA conversion/%	PBzMA DP	*V*_m_/nm^3^	*R*_out_/nm	*T*_m_/nm	*R*_in_/nm
58	72.3	108	27 ± 0.9	53 ± 18	10 ± 1.6	43 ± 18
60	75.4	113	28 ± 1.0	56 ± 19	11 ± 1.6	45 ± 19
62	78.2	117	29 ± 1.0	56 ± 19	11 ± 1.6	45 ± 19
64	80.7	122	30 ± 1.0	56 ± 19	12 ± 1.6	44 ± 19
68	85.2	128	32 ± 1.1	57 ± 20	12 ± 1.8	45 ± 20
72	88.9	134	33 ± 1.1	57 ± 20	13 ± 1.8	44 ± 20
76	91.8	138	34 ± 1.2	57 ± 19	13 ± 1.9	44 ± 19
88	97.1	146	36 ± 1.2	57 ± 20	14 ± 2.0	44 ± 20
120	100	150	37 ± 1.3	59 ± 20	14 ± 2.2	45 ± 21

Precise knowledge of the dimensions of the growing vesicles is important, because in principle this enables the vesicle growth mechanism to be deduced. For example, Warren *et al.*^67^ reported that the overall diameter of poly(glycerol monomethacrylate)–poly(2-hydroxypropyl methacrylate) (PGMA–PHPMA) vesicles prepared *via* RAFT aqueous dispersion polymerization remained constant while *T*_m_ increased when targeting higher PHPMA DPs. This indicates that the constrained vesicles grow inwards, with the thickening membrane leading to a reduction in the vesicle lumen volume. In order to elucidate the growth mechanism for the PSMA_13_–PBzMA_*x*_ vesicles described in this work, much higher PBzMA DPs must be targeted. Consequently, PSMA_13_–PBzMA_*x*_ vesicles with PBzMA DPs up to 2000 (prepared at 10% w/w solids on a 5.0 mL scale) were subjected to *post mortem* analysis using DLS, TEM and SAXS (see ESI†). DLS studies indicated that the overall vesicle diameter remained essentially constant (140–145 nm) for PBzMA DPs of between 100 and 400 (see Fig. 9c, blue data). For reference, the corresponding SAXS data reported in Table 3 for the *in situ* SAXS studies are also shown in Fig. 9c. The apparent discrepancy between these two data sets simply reflects the intensity-average and volume-average vesicle diameters reported by DLS and SAXS respectively. DLS diameters progressively increased for PBzMA DPs between 500 and 2000, while the corresponding size distributions significantly broadened for PBzMA DPs above 900. These data suggest that the vesicles become unstable for PBzMA DPs greater than 400, as similarly reported by Warren *et al.* for PGMA–PHPMA vesicles.^67^ TEM studies (see Fig. S8a†) support these DLS data: vesicles with narrow size distributions and approximately constant diameters were observed for PBzMA DPs up to 400. This indicates that the *apparent* modest increase in overall vesicle dimensions observed in the *in situ* SAXS studies (see Table 3) is actually an artefact. Moreover, the vesicle membrane thickness increases with PBzMA DP over this range, which suggests a similar ‘inward growth’ mechanism. Importantly, there is excellent agreement between the *in situ* and *post mortem T*_m_ data sets shown in Fig. 9b, which supports the validity of our kinetics renormalization approach. Furthermore, large, ill-defined species are observed by TEM for PBzMA DPs above 500 (see vertical dashed line in Fig. 9c). This is consistent with observations made by Warren *et al.*,^67^ who reported loss of the vesicular morphology for PHPMA DPs above 1000. In view of these observations, further *post mortem* SAXS studies were undertaken to monitor the evolution of the PSMA_13_–PBzMA_*x*_ morphology (see red data set in Fig. 9c and also S8b†). It should be noted that these additional SAXS measurements were performed using an in-house NanoStar instrument, rather than a synchrotron X-ray source. Thus the accessible *q* range was only sufficient to allow the evolution in *T*_m_ to be monitored; no information regarding the overall vesicle dimensions could be obtained. *T*_m_ increased monotonically from 9 nm to 30 nm on increasing the target PBzMA DP from 100 to 400. These data were fitted to the power law *T*_m_ = *kx*^*α*^ where *k* is a constant and *x* is the PBzMA DP. The *α* exponent was calculated to be 0.86, which is consistent with that reported by Warren *et al.*^67^ for *post mortem* SAXS analysis of PGMA–PHPMA vesicles (*α* = 0.79). For PBzMA DPs above 400, the *T*_m_ feature at around *q* = 0.2 nm^–1^ to 0.6 nm^–1^ becomes increasingly indistinct. This indicates the gradual loss of the vesicular morphology, which is consistent with the corresponding TEM studies. Since the DLS data indicate approximately the same overall vesicle dimensions for PBzMA DPs of 100–400, this indicates that the ‘inward growth’ mechanism is valid for both aqueous and non-polar media (see Fig. 9d). This is important, because it implies a *generic* vesicle growth mechanism for all PISA formulations. This is perfectly reasonable, because Warren *et al.* showed that this hitherto unrecognized mechanism is the *only* means by which the vesicles can lower their total surface area, and hence reduce their overall free energy.^67^

In the case of vesicles, different equations are required for the calculation of mean aggregation number per vesicle (*N*_v_), *S*_agg_ and *d*_int_, as indicated below (see eqn (4)–(6)). By definition, the volume fraction of BzMA *monomer* within the core domain (*φ*_BzMA_) at full conversion must be zero. Moreover, the SAXS data fits suggest that the volume fraction of *solvent* within the PBzMA chains forming the vesicle membrane (*x*_sol_) is close to zero. In this case, *N*_v_ for the final PSMA_13_–PBzMA_150_ vesicles can be calculated using eqn (4) below.4
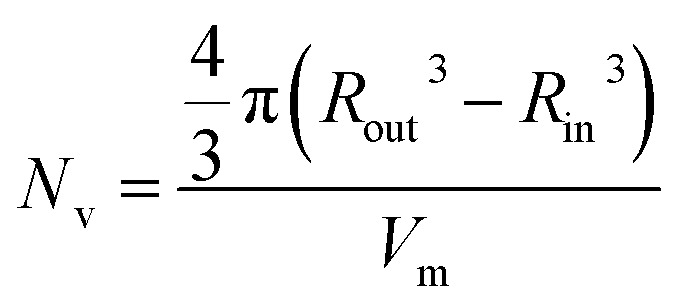



As for the earlier *in situ* SAXS studies conducted when targeting PSMA_31_–PBzMA_2000_ spheres, the leading error in the calculation of *N*_v_ is the MWD of the core-forming PBzMA block, which dictates the error in *V*_m_. From the GPC data obtained for PSMA_13_–PBzMA_150_ vesicles prepared on a laboratory scale, the standard deviation in *V*_m_ was estimated to be 3.4% using the same method used for the spheres (see ESI†). *S*_agg_ and *d*_int_ for the PSMA_13_–PBzMA_150_ vesicles are subsequently calculated using eqn (5) and (6), respectively.5
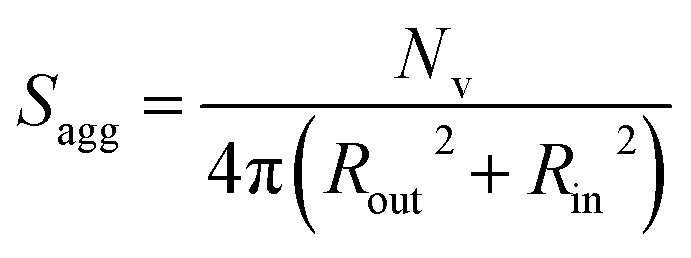

6
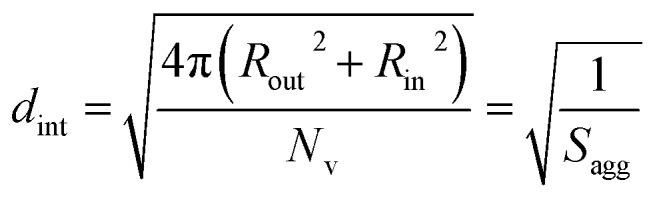



The *N*_v_ value calculated for PSMA_13_–PBzMA_150_ vesicles at full conversion was 12 700 ± 400, with the corresponding *S*_agg_ determined to be 0.187 ± 0.006 nm^–2^ and the average distance between adjacent copolymer chains at the core–shell interface (*d*_int_) was 2.31 ± 0.08 nm. These data somewhat differ to those calculated for related aqueous^67^ and alcoholic^48^ PISA formulations, where *d*_int_ is (retrospectively) calculated to be 3.1–3.4 nm. However, the solvent volume fraction within the vesicle membrane was found to be more than 0.35 in these earlier literature examples compared to essentially zero in the present work. This indicates that the copolymer chains are more densely packed in the current non-polar PISA formulation. Notably, the value of *d*_int_ calculated for these vesicles is comparable to that determined for densely-packed polybutadiene–poly(l-lysine) block copolymer chains within vesicle membranes formed in saline solution (*d*_int_ = 2.4 nm at pH 10.3).^89^ The *S*_agg_ and *d*_int_ values calculated for PSMA_13_–PBzMA_150_ vesicles can also be compared to those for PSMA_31_–PBzMA_2000_ spheres (*S*_agg_ = 0.039 ± 0.004 nm^–2^, *d*_int_ = 5.04 ± 0.48 nm). It is evident that the copolymer chains are packed more densely within the PSMA_13_–PBzMA_150_ vesicles compared to the PSMA_31_–PBzMA_2000_ spheres. This is likely to be the result of the differing interfacial curvatures associated with each copolymer morphology, but the significant difference in target DP for the core-forming PBzMA blocks may also be a factor.

## Conclusions

In summary, a range of sterically-stabilized PSMA–PBzMA diblock copolymer nano-objects have been prepared *via* RAFT dispersion polymerization in mineral oil. Improved control over the copolymer molecular weight distribution is achieved compared to previously reported PISA syntheses conducted in non-polar media, with relatively narrow molecular weight distributions (*M*_w_/*M*_n_ ≤ 1.30) being achieved even when targeting PBzMA DPs of up to 500. As expected, only spherical nanoparticles were obtained when using relatively long PSMA_18_ or PSMA_31_ macro-CTAs. In both cases, a log–log plot indicated a linear correlation between the mean sphere diameter (as judged by DLS) and the core-forming PBzMA DP. PSMA_31_–PBzMA_*x*_ spheres indicated a scaling exponent of 0.50, suggesting essentially non-solvated PBzMA chains within the core-forming PBzMA block, whereas a scaling exponent of 0.61 was obtained for PSMA_18_–PBzMA_*x*_ spheres, suggesting a finite degree of solvation for the PBzMA chains in this case. In contrast, using a relatively short PSMA_13_ macro-CTA allows the synthesis of spherical, worm-like or vesicular morphologies. Construction of a detailed phase diagram for PSMA_13_–PBzMA_*x*_ diblock copolymers confirmed that pure spheres, worms or vesicles could be obtained at relatively low solids concentrations. This is important, because it facilitates *in situ* SAXS studies of the formation of PSMA_31_–PBzMA_2000_ spheres and PSMA_13_–PBzMA_150_ nano-objects at 10% w/w solids. However, the rate of BzMA polymerization during such scattering experiments is significantly faster than that observed under normal laboratory conditions. Thus the latter kinetic data sets require renormalization to enable detailed analysis of the *in situ* SAXS data. When targeting PSMA_31_–PBzMA_2000_ spheres, a systematic increase in core diameter (*D*_s_) and mean aggregation number (*N*_s_) are clearly discernible during the BzMA polymerization, with the final scattering pattern indicating the formation of near-monodisperse PSMA_31_–PBzMA_2000_ spheres. Interestingly, the number of copolymer chains per unit surface area (*S*_agg_) decreased rapidly during the initial stages of the polymerization until a limiting value of ∼0.038 nm^–2^ is attained. This indicated that the mean distance between copolymer chains at the core–shell interface (*d*_int_) at full conversion was approximately 5.0 nm. When targeting PSMA_13_–PBzMA_150_ vesicles, characteristic scattering patterns for the dissolved copolymer chains, intermediate spheres and worms, and the final vesicle morphology were obtained. Importantly, revisiting the phase diagram constructed for this formulation enabled validation of the renormalization protocol adopted for analysis of the kinetic data. More specifically, the mean PBzMA DPs corresponding to the various phase boundaries were in relatively good agreement with the upper and lower DPs assigned to the corresponding pure phases indicated by analysis of the *in situ* SAXS patterns. Within the mixed phase space, it was shown that vesicles are formed from worms *via* octopi and jellyfish intermediates as first reported for an aqueous PISA formulation. Combined DLS, TEM and SAXS studies indicate that the overall vesicle dimensions remain relatively constant as the vesicle membrane gradually thickens with increasing PBzMA DP until so-called vesicle ‘death’ (or break-up) occurs. These observations indicate an ‘inward growth’ mechanism, as recently reported for an aqueous PISA formulation. This suggests that a *generic* vesicle growth mechanism is most likely applicable for PISA syntheses.

## Supplementary Material

Supplementary information
