# Supplementary material for: In situ small-angle X-ray scattering studies of sterically-stabilized diblock copolymer nanoparticles formed during polymerization-induced self-assembly in non-polar media
Source: Chem Sci. 2016 Apr 18;7(8):5078–90. doi: 10.1039/c6sc01243d (PMC6018718; doi:10.1039/c6sc01243d)
Supplement: Supplementary file 1 [file SC-007-C6SC01243D-s001.pdf]

Electronic Supplementary Material (ESI) for Chemical Science.
This journal is © The Royal Society of Chemistry 2016

Supporting Information for:

In situ small-angle X-ray scattering studies of sterically-stabilized diblock copolymer nanoparticles formed during polymerization-induced self-assembly in non-polar media

Matthew J. Derry,*[,a] Lee A. Fielding,[a,b] Nicholas J. Warren,[a] Charlotte J. Mable,[a] Andrew J. Smith,[c] Oleksandr O. Mykhaylyk*[,a] and Steven P. Armes*[,a]

[a] Dainton Building, Department of Chemistry, The University of Sheffield, Brook Hill, Sheffield, South Yorkshire, S3 7HF, UK.

[b] Present address: The School of Materials, The University of Manchester, Oxford Road, Manchester, M13 9PL, UK.

[c] Diamond Light Source Ltd., Diamond House, Harwell Science and Innovation Campus, Didcot, Oxfordshire, OX11 0DE, UK.

Experimental Section

Materials

Monomers were purchased from Sigma-Aldrich (UK) and passed through a basic alumina column prior to use to remove inhibitor. Tert-butyl peroxy-2-ethylhexanoate (T21s) initiator was purchased from AkzoNobel (The Netherlands). Cumyl dithiobenzoate (CDB), $CDCl_3$, and all other reagents were purchased from Sigma-Aldrich (UK) and were used as received, unless otherwise noted. THF, *n*-heptane and toluene were purchased from Fisher Scientific (UK), CD_2Cl_2 was purchased from Goss Scientific (UK) and industrial-grade mineral oil was provided by Lubrizol Corporation Ltd. Each of these solvents was used as received.

Synthesis of poly(stearyl methacrylate) macro-chain transfer agent via RAFT solution polymerization

A typical synthesis of $PSMA_{31}$ macro-CTA was conducted as follows. A 250 mL round-bottomed flask was charged with stearyl methacrylate (SMA; 37.3 g; 110 mmol), cumyl dithiobenzoate (CDB; 1.00 g; 3.67 mmol; target degree of polymerization = 30), 2,2'-azobisisobutyronitrile (AIBN; 121 mg, 0.74 mmol; CDB/AIBN molar ratio = 5.0) and toluene (57.5 g). The sealed reaction vessel was purged with nitrogen and placed in a pre-heated oil bath at 70 °C for 10 h. The resulting PSMA (SMA conversion = 72 %; M_n = 9,200 g mol^{-1}, M_w = 11,100 g mol^{-1}, M_w/M_n = 1.21) was purified by precipitation into excess ethanol. The mean degree of polymerization (DP) of this macro-CTA was calculated to be 31 using ^{1}H NMR spectroscopy by comparing the integrated signals corresponding to the CDB aromatic protons at 7.0-7.5 ppm with that assigned to the two oxymethylene protons of PSMA at 3.4-4.2 ppm. Thus the CTA efficiency was calculated to be 70%.

Synthesis of poly(stearyl methacrylate)-poly(benzyl methacrylate) diblock copolymer nanoparticles via RAFT dispersion polymerization

A typical RAFT dispersion polymerization synthesis of $PSMA_{31}$-$PBzMA_{196}$ diblock copolymer nanoparticles at 20 % w/w solids was conducted as follows. Benzyl methacrylate (BzMA; 0.393 g; 2.23 mmol), T21s initiator (4.82 mg; 2.23 μmol; dissolved at 10.0 % v/v in mineral oil) and $PSMA_{31}$ macro-CTA (0.12 g; 11.1 μmol; macro-CTA/initiator molar ratio = 5.0; target degree of polymerization of PBzMA = 200) were dissolved in mineral oil (2.05 g). The reaction mixture was sealed in a 10 mL round-bottomed flask and purged with nitrogen gas for 30 min. The deoxygenated solution was then placed in a pre-heated oil bath at 90 °C for 5 h (final BzMA conversion = 98 %; M_n = 30,100 g mol^{-1}, M_w/M_n = 1.19).

Gel permeation chromatography

Molecular weight distributions were assessed by gel permeation chromatography (GPC) using THF eluent. The THF GPC set-up comprised two 5 μm (30 cm) Mixed C columns and a WellChrom K-2301 refractive index detector operating at 950 ± 30 nm. The mobile phase contained 2.0% v/v triethylamine and 0.05 w/v % butylhydroxytoluene (BHT) and the flow rate was 1.0 mL min^{-1}. A series of ten near-monodisperse poly(methyl methacrylate) standards (M_p values ranging from 645 to 2,480,000 g mol^{-1}) were used for calibration.

^{1}H NMR spectroscopy

Spectra were recorded in either CD_2Cl_2 or $CDCl_3$ using a Bruker AV1-400 or AV1-250 MHz spectrometer. Typically 64 scans were averaged per spectrum.

Dynamic light scattering

Dynamic light scattering (DLS) studies were performed at 25 °C using a Zetasizer NanoZS instrument (Malvern Instruments, UK) at a fixed scattering angle of 173°. Copolymer dispersions were diluted to 0.10% w/w using *n*-heptane prior to light scattering studies. The intensity-average diameter and polydispersity of the diblock copolymer nanoparticles were calculated by cumulants analysis of the experimental correlation function using Dispersion Technology Software version 6.20. Data were averaged over thirteen runs each of thirty seconds duration.

Transmission electron microscopy

Transmission electron microscopy (TEM) studies were conducted using a Philips CM 100 instrument operating at 100 kV and equipped with a Gatan 1 k CCD camera. Diluted diblock copolymer solutions (0.10 % w/w) were placed as droplets on carbon-coated copper grids and exposed to ruthenium(VIII) oxide vapor for 7 min at 20 °C and dried prior to analysis.[1] This heavy metal compound acted as a positive stain for the core-forming PBzMA block in order to improve contrast. The ruthenium(VIII) oxide was prepared as follows: ruthenium(IV) oxide (0.30 g) was added to water (50 g) to form a black slurry; addition of sodium periodate (2.0 g) with stirring produced a yellow solution of ruthenium(VIII) oxide within 1 min.

Small-angle X-ray scattering

SAXS patterns were collected at a synchrotron source (Diamond Light Source, station I22, Didcot, UK) using monochromatic X-ray radiation (wavelength, λ = 0.124 nm, with q ranging from 0.015 to 1.3 nm^{-1}, where $q = 4\pi \sin \theta/\lambda$ is the length of the scattering vector and θ is one-

half of the scattering angle) and a 2D Pilatus 2M pixel detector (Dectris, Switzerland). Glass capillaries of 2.0 mm diameter were used as a sample holder. For *in situ* SAXS studies, all reagents were first purged with nitrogen gas for 30 min, as described earlier, before a portion of the deoxygenated solution was transferred into a glass capillary. The capillary was then sealed in order to prevent exposure to oxygen before being placed into the brass holding stage, which was pre-heated to 90 °C using a water circulating bath. SAXS patterns were collected every 2 min for 3 h, or until no further evolution in the pattern was observed. SAXS data were reduced (integration, normalization and absolute intensity calibration using SAXS patterns of deionized water assuming that the differential scattering cross-section of water is 0.0162 cm^{-1}) using Dawn software supplied by Diamond Light Source.[2]

Selected static SAXS patterns were obtained for 1.0% w/w copolymer dispersions using a Bruker AXS Nanostar instrument modified with microfocus X-ray tube (GeniX3D, Xenocs) and motorized scatterless slits for the beam collimation (sample to detector distance 1.46 m, Cu Kα radiation and 2D HiSTAR multiwire gas detector). SAXS patterns were recorded over a q range of 0.08 $nm^{-1} < q < 1.6$ nm^{-1}. Glass capillaries of 2.0 mm diameter were used as a sample holder, and an exposure time of 1.0 h was utilized for each sample. SAXS data were reduced using Nika macros for Igor Pro by J. Ilavsky. All SAXS data collected at different locations were analyzed (background subtraction, data modelling and fitting) using Irena SAS macros for Igor Pro.[3]

Renormalization of kinetic data for the RAFT dispersion polymerization of BzMA

Polymerization kinetic data were obtained for normal 10 mL laboratory-scale PISA syntheses (targeting $PSMA_{31}$-$PBzMA_{2000}$ spheres and $PSMA_{13}$-$PBzMA_{150}$ vesicles, respectively) by withdrawing multiple aliquots of the reaction solution prior to ^{1}H NMR analysis (see Figure 4a and Figure S3; blue data sets in each case). In each case these data were fitted to a sigmoid function using Igor Pro software using equation S1 shown below:

$$y = a + \frac{b}{\left(1 + exp\left(\frac{c - x}{d}\right)\right)} \tag{S1}$$

Here *y* is the BzMA conversion (%), *x* is the relative polymerization time and *a*, *b*, *c* and *d* are arbitrary fitting parameters. This function was then utilized to calculate the polymerization kinetics for the two PISA syntheses conducted in a 2.0 mm glass capillary for the *in situ* SAXS experiments described above (see Figure 4a and Figure S3; red data sets in each case).

Determination of BzMA volume fraction in $PSMA_{31}$-$PBzMA_x$ spherical nanoparticle cores

$PSMA_{31}$-$PBzMA_x$ spheres prepared at 20 % w/w solids in mineral oil with x values of 396, 582, 784 or 1470 were diluted to 10% w/w in the same solvent. Then the relevant amounts of BzMA monomer (4-170 μL) and additional mineral oil were added to 2.0 mL aliquots of the above dispersion in order to replicate various time points during the RAFT dispersion polymerization of $PSMA_{31}$-$PBzMA_{2000}$ spheres that correspond to BzMA conversions of 19.8%, 29.1%, 39.2% or 73.5%, respectively. Each BzMA-doped dispersion was then heated to 90 °C for 1 h before being sedimented using a Heraeus Biofuge Pico centrifuge at 13,000

rpm (16,060 g) until the spheres were fully sedimented. The resulting clear supernatant, which contains any BzMA monomer not located within the nanoparticle cores, was removed and analyzed *via* ^{1}H NMR spectroscopy in CD_2Cl_2 using triethoxymethylsilane (TEMS) as an internal standard (present at the same concentration as the BzMA monomer prior to centrifugation). The integrated oxymethylene signal due to the TEMS (~ 3.8 ppm) was set to six protons and the NMR signals corresponding to the aromatic protons ([Ar]) of the BzMA monomer were then integrated. The *mole fraction* of BzMA monomer present within the nanoparticle cores is therefore equal to 1-([Ar]/5). The BzMA *volume fraction* within the core domain (φ_{BzMA}) was subsequently calculated by considering the relative volumes of the monomer (as calculated using ^{1}H NMR spectroscopy; see Figure S1 below for the calibration plot) and the PBzMA core-forming chains within the nanoparticle cores. Given that 100% BzMA conversion corresponds to $\varphi_{BzMA} = 0$, these data can be used to calculate φ_{BzMA} at any time point during the PISA synthesis of $PSMA_{31}$-$PBzMA_{2000}$ spheres. A plot of BzMA conversion (x) vs. φ_{BzMA} (y) gave a satisfactory fit ($R^2 > 0.95$) to a logarithmic function of the form:

$y = -0.234*\ln(x) + 1.0656$

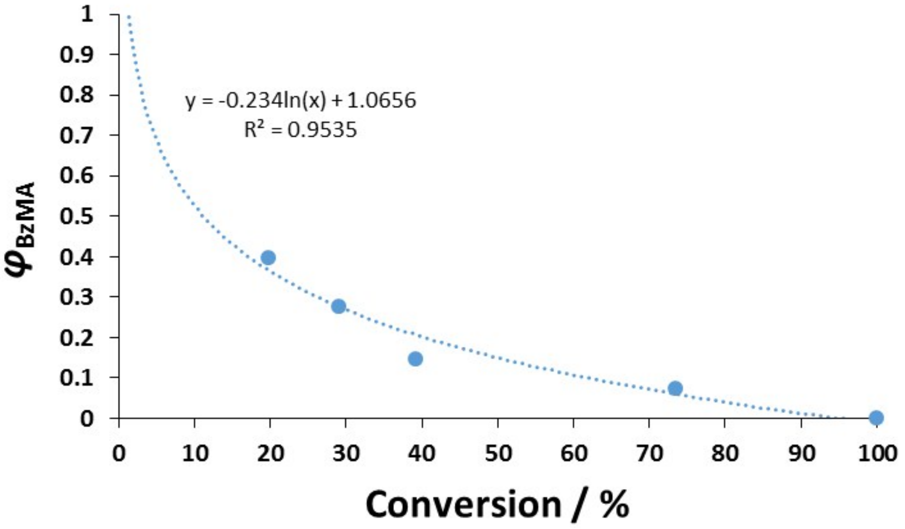

Figure S1. Conversion vs. volume fraction of BzMA monomer within growing spherical cores (φ_{BzMA}) calculated for the PISA synthesis of $PSMA_{31}$-$PBzMA_{2000}$ spheres at 10% w/w.

Determination of the standard deviation in the molecular weight distribution (MWD)

The standard deviation in the MWD is required in order to determine the maximum error that should be attributed to the number of copolymer chains per self-assembled sphere or vesicle (N_{agg}). This is because the dominant error in this calculation comes from the uncertainty in the mean volume occupied by one PBzMA core-forming block (V_{PBzMA}), which is in turn determined by the MWD. Therefore the unimodal MWD determined by THF GPC analysis (see Figure S2) was fitted to a Gaussian model to determine its standard deviation using Equation S2 below:

$$y = a\,exp\left(-\frac{(x-b)^2}{2\sigma^2}\right) \qquad \text{(S2)}$$

Here y is the retention time (min), x is the detector response, a and b are constants and σ is the standard deviation. This σ value corresponded to either 9.5% or 3.4% of the peak retention time for $PSMA_{13}$-$PBzMA_{2000}$ and $PSMA_{13}$-$PBzMA_{150}$ diblock copolymers respectively. This parameter was subsequently used as the maximum percentage error for the relevant N_{agg} calculations.

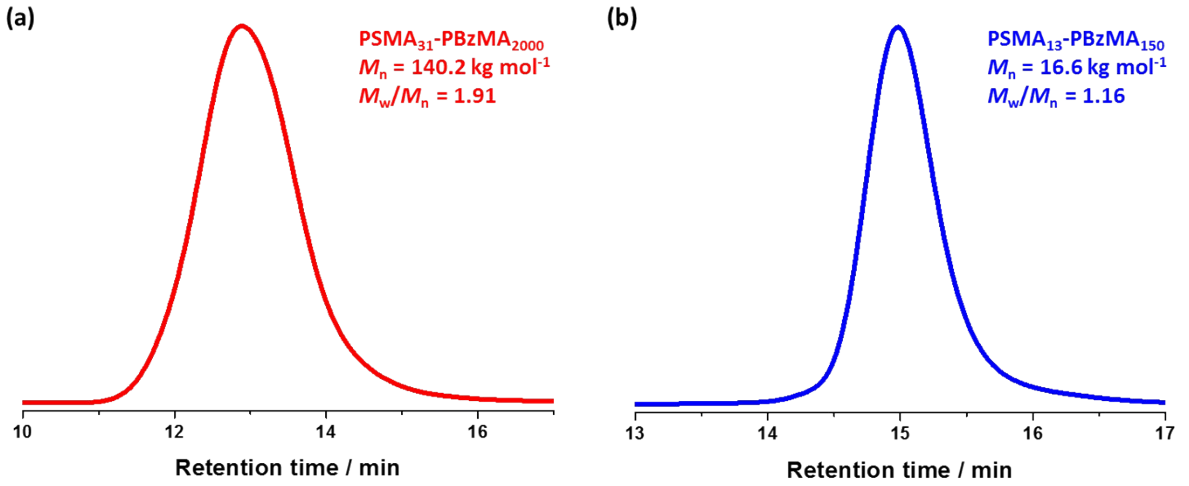

Figure S2. Gel permeation chromatograms used for the determination of the standard deviation in the molecular weight distributions during the PISA syntheses of (a) $PSMA_{31}$-$PBzMA_{2000}$ spheres and (b) $PSMA_{13}$-$PBzMA_{150}$ vesicles at 10% w/w.

Supplementary Tables and Figures

Table S1. Summary of monomer conversions, mean degrees of polymerization and GPC molecular weights for three PSMA macro-CTAs prepared by RAFT solution polymerization of SMA in toluene at 70 °C using AIBN and CDB. Conditions: total solids concentration = 40% w/w solids, [CDB]/[AIBN] molar ratio = 5.0.

Target DP	^{1}H NMR Conversion %	Actual DP by ^{1}H NMR	THF GPC		
			M_n / g mol^{-1}	M_w / g mol^{-1}	M_w/M_n
$PSMA_{30}$	72	31	9,200	11,100	1.21
$PSMA_{10}$	75	18	5,500	6,900	1.24
$PSMA_5$	76	13	4,900	5,700	1.17

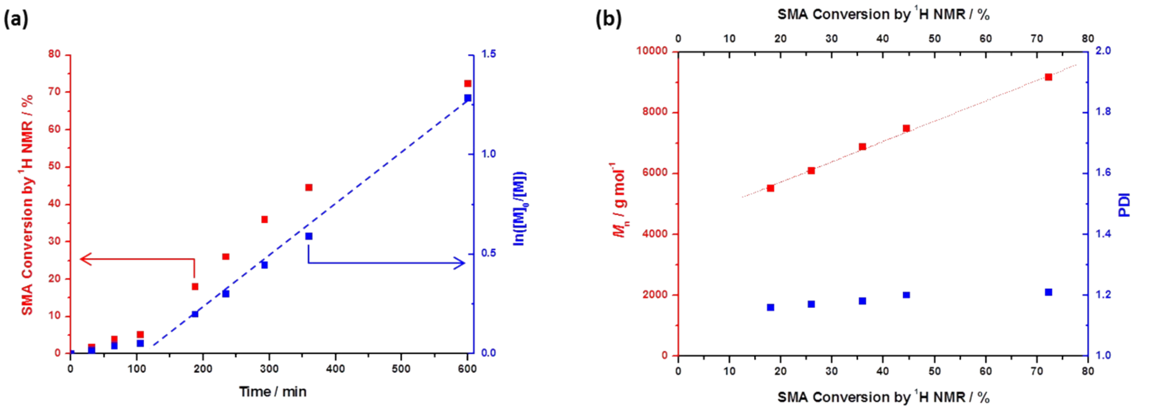

Figure S3. RAFT solution polymerization of SMA in toluene at 70 °C using AIBN initiator and CDB CTA targeting a DP of 30: (a) conversion vs. time curve and corresponding semi-logarithmic plot; (b) M_n and M_w/M_n vs. conversion plots. Conditions: total solids concentration = 40% w/w, [CDB]/[AIBN] molar ratio = 5.0.

Table S2. Summary of targeted copolymer composition, BzMA conversions, GPC molecular weights, DLS data and observed TEM morphology for two series of $PSMA_{31}$-$PBzMA_x$ and $PSMA_{18}$-$PBzMA_x$ diblock copolymers prepared by RAFT dispersion polymerization of BzMA in mineral oil at 90 °C using T21s initiator. Conditions: [PSMA macro-CTA]/[T21s] molar ratio = 5.0.

**Target Composition**	**Total Solids Concentration / % w/w**	**% BzMA**	**THF GPC**		**DLS**		**TEM Morphology**
			M_n	M_w / M_n	**d / nm**	**PDI**	
$PSMA_{31}$-$PBzMA_{50}$	20	99	14,300	1.20	25	0.04	Spheres
$PSMA_{31}$-$PBzMA_{100}$	20	98	18,300	1.23	34	0.02	Spheres
$PSMA_{31}$-$PBzMA_{200}$	20	98	30,100	1.19	44	0.01	Spheres
$PSMA_{31}$-$PBzMA_{300}$	20	99	38,000	1.25	54	0.01	Spheres
$PSMA_{31}$-$PBzMA_{400}$	20	99	51,400	1.22	62	0.01	Spheres
$PSMA_{31}$-$PBzMA_{500}$	20	99	55,700	1.30	67	0.07	Spheres
$PSMA_{31}$-$PBzMA_{600}$	20	97	66,800	1.36	82	0.16	Spheres
$PSMA_{31}$-$PBzMA_{800}$	20	98	86,300	1.40	101	0.18	Spheres
$PSMA_{31}$-$PBzMA_{1000}$	20	99	113,400	1.42	114	0.17	Spheres
$PSMA_{31}$-$PBzMA_{1500}$	20	98	132,200	1.56	125	0.07	Spheres
$PSMA_{31}$-$PBzMA_{2000}$	20	98	140,200	1.91	154	0.01	Spheres
$PSMA_{18}$-$PBzMA_{50}$	20	99	9,000	1.26	23	0.02	Spheres
$PSMA_{18}$-$PBzMA_{75}$	20	99	11,600	1.25	39	0.08	Spheres
$PSMA_{18}$-$PBzMA_{100}$	20	98	15,800	1.25	41	0.01	Spheres
$PSMA_{18}$-$PBzMA_{150}$	20	98	18,600	1.24	48	0.02	Spheres
$PSMA_{18}$-$PBzMA_{200}$	20	98	23,300	1.24	58	0.03	Spheres
$PSMA_{18}$-$PBzMA_{400}$	20	98	42,700	1.26	93	0.02	Spheres
$PSMA_{18}$-$PBzMA_{500}$	20	98	48,800	1.34	114	0.04	Spheres
$PSMA_{18}$-$PBzMA_{800}$	20	98	69,200	1.41	135	0.01	Spheres

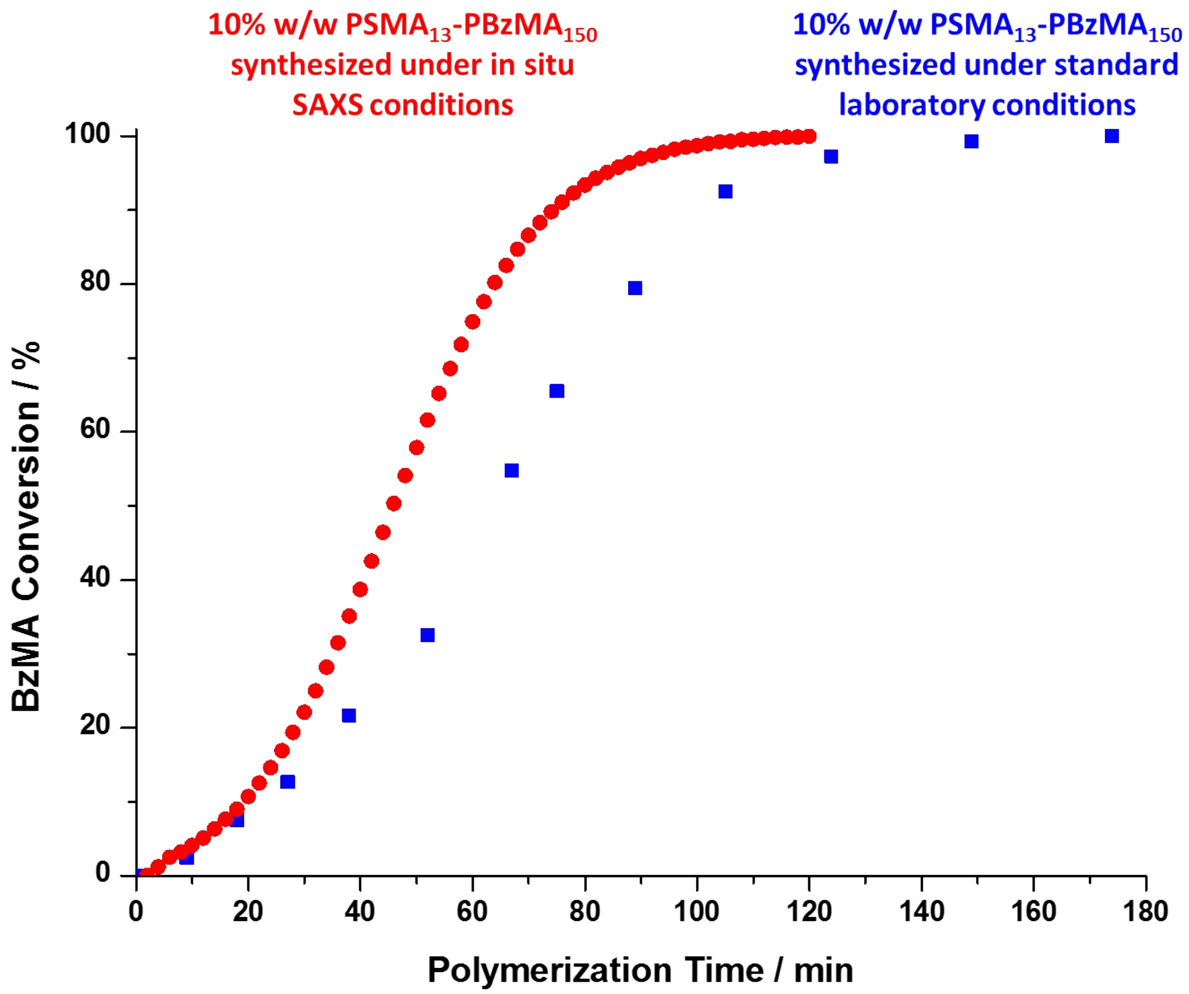

Figure S4. Conversion vs. time curve (blue squares) for the RAFT dispersion polymerization of BzMA in mineral oil at 90 °C when targeting $PSMA_{13}$-$PBzMA_{150}$ block copolymer vesicles at 10% w/w solids using T21s initiator under normal laboratory conditions and the renormalized conversion vs. time curve (red circles) calculated for the same PISA synthesis conducted using the same formulation during in situ SAXS studies.

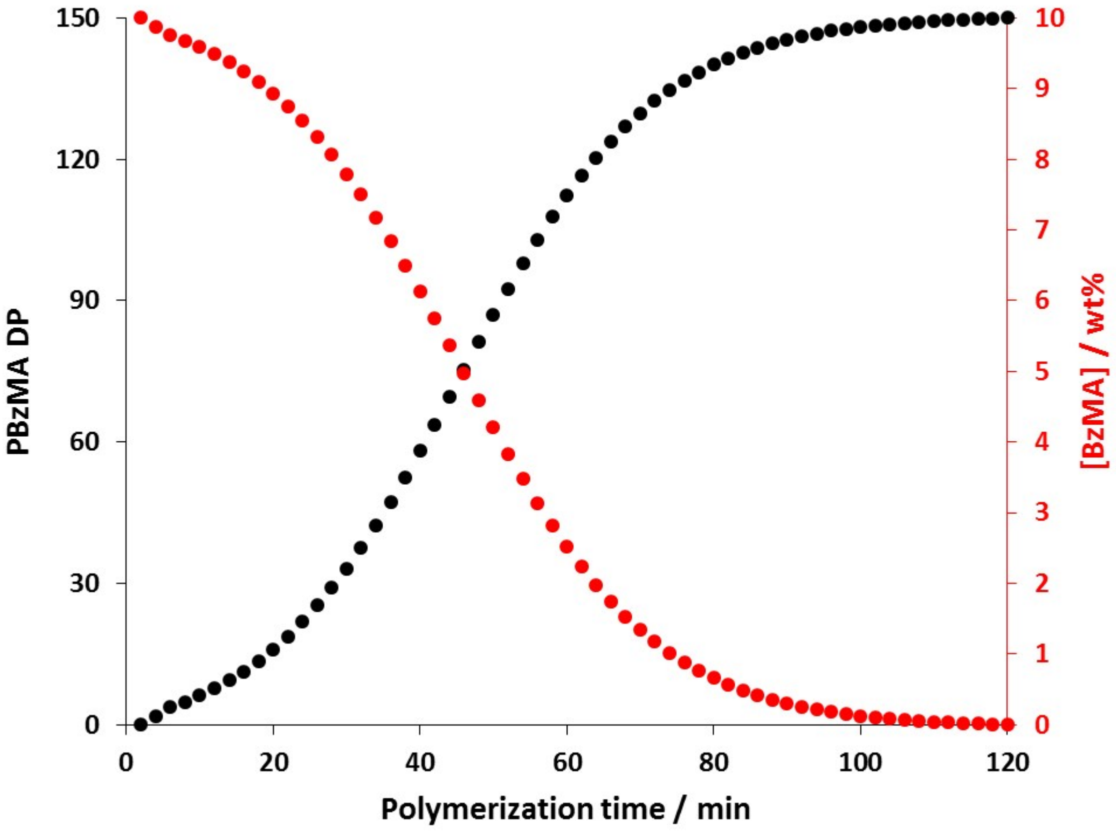

Figure S5. Change in the PBzMA DP (black data) and the concentration of BzMA monomer ([BzMA], red data) during the *in situ* SAXS studies when targeting $PSMA_{13}$-$PBzMA_{150}$ vesicles.

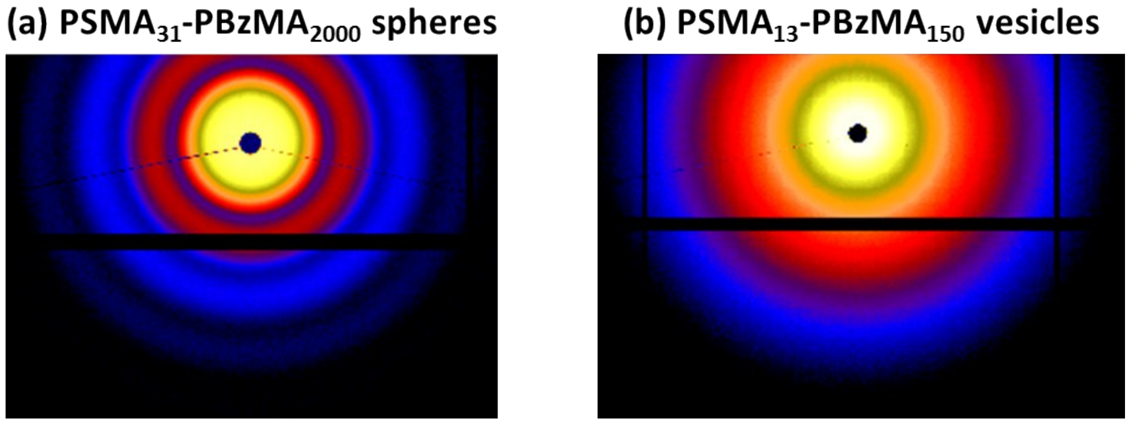

Figure S6. 2D scattering patterns obtained at the end of the *in situ* SAXS studies for the PISA synthesis of (a) $PSMA_{31}$-$PBzMA_{2000}$ spheres and (b) $PSMA_{13}$-$PBzMA_{150}$ vesicles at 10% w/w.

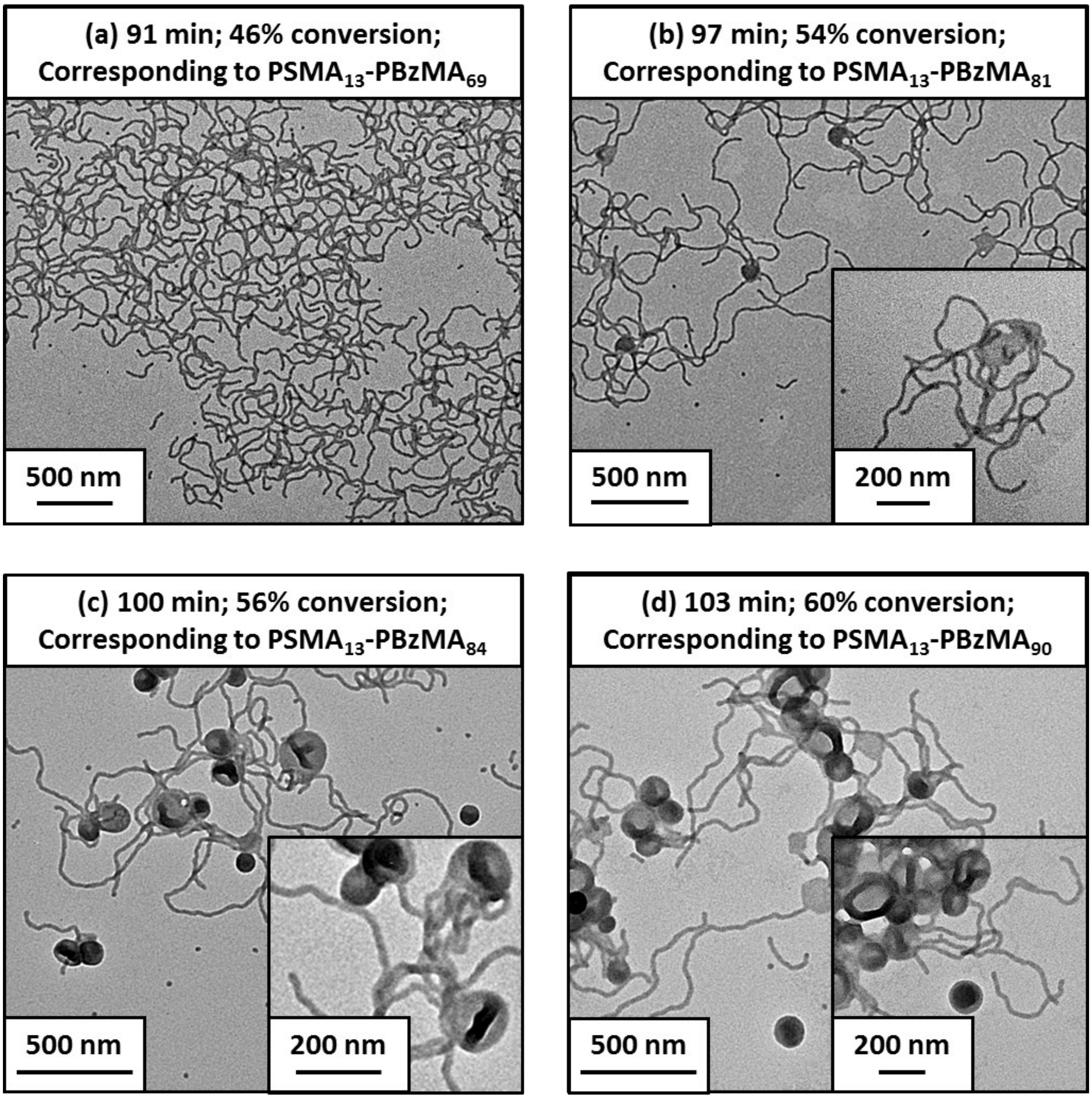

Figure S7. Transmission electron micrographs obtained for 0.1% w/w dispersions of $PSMA_{13}$-$PBzMA_x$ nanoparticles obtained at various time points during the PISA synthesis of $PSMA_{13}$-$PBzMA_{150}$ vesicles under standard laboratory conditions at 10% w/w in mineral oil. (a) A pure worm morphology is observed after 91 min, (b) worms and octopi structures are observed after 97 min and worms, vesicles, octopi and jellyfish structures are observed after (c) 100 min and (d) 103 min.

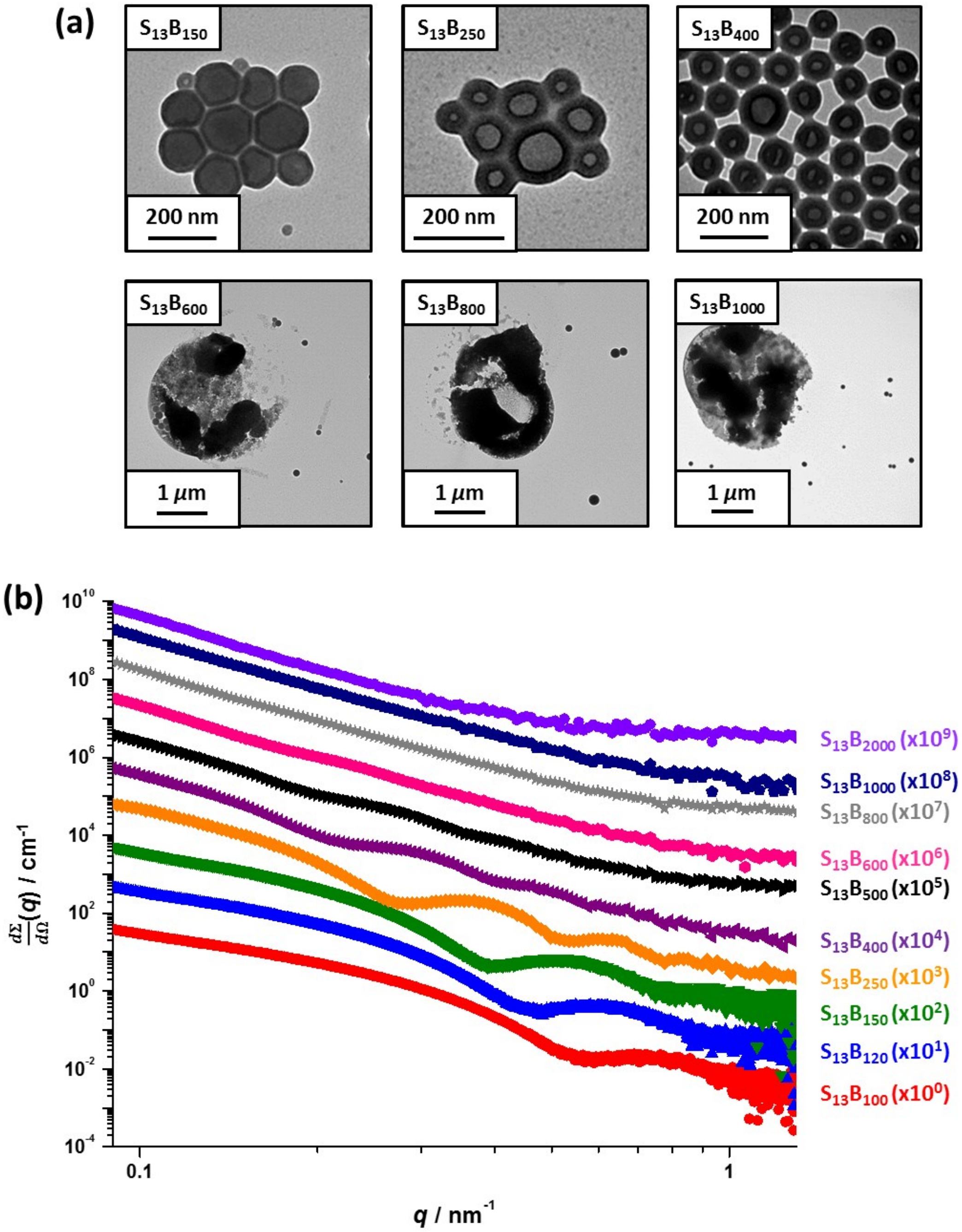

Figure S8. (a) Transmission electron micrographs obtained for 0.1% w/w dispersions, and (b) SAXS patterns obtained for 1.0% w/w dispersions, of selected $PSMA_{13}$-$PBzMA_x$ (denoted $S_{13}B_x$) nanoparticles.

SAXS models

In general, the intensity of X-rays scattered by a dispersion of nano-objects [usually represented by the scattering cross section per unit sample volume, $\frac{d\Sigma}{d\Omega}(q)$] can be expressed as:

$$\frac{d\Sigma}{d\Omega}(q) = NS(q)\int_0^{\infty}...\int_0^{\infty} F(q,r_1,...,r_k)^2\Psi(r_1,...,r_k)dr_1...dr_k \quad \text{(S3)}$$

where $F(q,r_1,...,r_k)$ is the form factor, $r_1,...,r_k$ is a set of k parameters describing the structural morphology, $\Psi(r_1,...,r_k)$ is the distribution function, S(q) is the structure factor and N is the nano-object number density per unit volume expressed as:

$$N = \frac{\varphi}{\int_0^{\infty}...\int_0^{\infty} V(r_1,...,r_k)\Psi(r_1,...,r_k)dr_1...dr_k} \quad \text{(S4)}$$

where $V(r_1,...,r_k)$ is volume of the nano-object and φ is their volume fraction in the dispersion.

Spherical micelle model

The spherical micelle form factor for Equation (S3) is given by:[4]

$$F_{s\_mic}(q) = N_s^2\beta_s^2A_s^2(q,R_s) + N_s\beta_c^2F_c(q,R_g) + (q) \quad \text{(S5)}$$

where R_s is the core radius of the spherical micelle, R_g is the radius of gyration of the PSMA corona block. The core block and the corona block X-ray scattering length contrast is given by $\beta_s = V_s(\xi_s - \xi_{sol})$ and $\beta_c = V_c(\xi_c - \xi_{sol})$, respectively. Here ξ_s, ξ_c and ξ_{sol} are the X-ray scattering length densities of the core block ($\xi_{PBzMA} = 10.38 \times 10^{10}$ cm^{-2}), the corona block ($\xi_{PSMA} = 9.24 \times 10^{10}$ cm^{-2}) and the solvent ($\xi_{sol} = 7.63 \times 10^{10}$ cm^{-2}), respectively. V_s and V_c are volumes of the core block (V_{PBzMA}) and the corona block (V_{PSMA31}), respectively. The volumes were obtained from $V = \frac{M_{n,pol}}{N_A\rho}$ using the density of PBzMA (ρ_{PBzMA} = 1.15 g cm^{-3})[5] and the solid-state

homopolymer density of PSMA determined by helium pycnometry (ρ_{PSMA} = 0.97 g cm^{-3}), where $M_{n,pol}$ corresponds to the number-average molecular weight of the block determined by ^{1}H NMR spectroscopy. The sphere form factor amplitude is used for the amplitude of the core self-term:

$$A_c(q,R_s) = \Phi(qR_s)exp\left(-\frac{q^2\sigma^2}{2}\right) \tag{S6}$$

where $\Phi(qR_s) = \frac{3[\sin(qR_s) - qR_s cos(qR_s)]}{(qR_s)^3}$. A sigmoidal interface between the two blocks was assumed for the spherical micelle form factor [Equation (S6)]. This is described by the exponent term with a width σ accounting for a decaying scattering length density at the micellar interface. This σ value was fixed at 2.5 during fitting.

The form factor amplitude of the spherical micelle corona is:

$$A_c(q) = \frac{\int_{R_s}^{R_s+2s} \mu_c(r)\frac{sin(qr)}{qr}r^2dr}{\int_{R_s}^{R_s+2s} \mu_c(r)r^2dr}exp\left(-\frac{q^2\sigma^2}{2}\right) \tag{S7}$$

The radial profile, $\mu_c(r)$, can be expressed by a linear combination of two cubic b splines, with two fitting parameters s and a corresponding to the width of the profile and the weight coefficient, respectively. This information can be found elsewhere,[6, 7] as can the approximate integrated form of Equation (S7). The self-correlation term for the corona block is given by the Debye function:

$$F_c(q,R_g) = \frac{2\left[\exp\left(-q^2{R_g}^2\right) - 1 + q^2{R_g}^2\right]}{q^4{R_g}^4} \tag{S8}$$

where R_g is the radius of gyration of the PSMA coronal block. The aggregation number of the spherical micelle is:

$$N_s = \left(1 - x_{sol}\right)\frac{\frac{4}{3}\pi R_s^{\,3}}{V_s} \tag{S9}$$

where $x_{\rm sol}$ is the volume fraction of solvent in the PBzMA micelle core. An effective structure factor expression proposed for interacting spherical micelles[8] has been used in Equation (S3):

$$S_s(q) = 1 + \frac{A_{s\_mic}^{av}(q)^2\left[S_{PY}\left(q,R_{PY},f_{PY}\right) - 1\right]}{F_{s\_mic}(q)} \tag{S10}$$

Herein the form factor of the average radial scattering length density distribution of micelles is used as $A_{s\_mic}^{av}(q) = N_s\left[\beta_s A_s(q,R_s) + \beta_c A_c(q)\right]$ and $S_{PY}(q,R_{PY},f_{PY})$ is a hard-sphere interaction structure factor based on the Percus-Yevick approximation,[9] where $R_{\rm PY}$ is the interaction radius and $f_{\rm PY}$ is the hard-sphere volume fraction. A polydispersity for one parameter ($R_{\rm s}$) is assumed for the micelle model which is described by a Gaussian distribution. Thus, the polydispersity function in Equation (S3) can be represented as:

$$\Psi(r_1) = \frac{1}{\sqrt{2\pi\sigma_{Rs}^{\,2}}}exp\left(-\frac{\left(r_1 - R_s\right)^2}{2\sigma_{Rs}^{\,2}}\right) \tag{S11}$$

where σ_{Rs} is the standard deviation for $R_{\rm s}$. In accordance with Equation (S4), the number density per unit volume for the micelle model is expressed as:

$$N = \frac{\varphi}{\int_0^{\infty} V(r_1)\Psi(r_1)dr_1} \tag{S12}$$

where φ is the total volume fraction of copolymer in the spherical micelles and $V(r_1)$ is the total *volume* of copolymer in a spherical micelle $\left[V(r_1) = (V_s + V_c)N_s(r_1)\right]$.

The model fitting to the final SAXS pattern of PSMA$_{31}$-PBzMA$_{2000}$ spheres indicated φ = 0.063, $R_{\rm PY}$ = 62.8 nm and $f_{\rm PY}$ = 0.073, which is consistent with the expected volume fraction of polymer (0.075) in this system after synthesis at full conversion. The experimental $R_{\rm g}$

obtained from this fitting for the corona PSMA block (1.5 nm) is also physically reasonable, since it is close to the estimated parameter. Assuming that the contour length of a PSMA monomer is 0.255 nm (two C-C bonds in *all-trans* conformation), the total contour length of a $PSMA_{31}$ block, L_{PSMA31} = 31 × 0.255 nm = 7.905 nm. Given a mean Kuhn length of 1.53 nm [based on the known literature value for PMMA[10]] an estimated unperturbed radius of gyration, $R_g = (7.905 \times 1.53/6)^{0.5}$, or 1.42 nm is determined.

Vesicle model

The vesicle form factor in Equation (S3) is expressed as:[11]

$$F_{ves}(q) = N_v^2 \beta_m^2 A_m^2(q) + N_v \beta_{vc}^2 F_c(q,R_g) + N_v(\text{[illegible]} (q) \qquad \text{(S13)}$$

The X-ray scattering length contrast for the membrane-forming block (PBzMA) and the coronal stabiliser block (PSMA) is given by $\beta_m = V_m(\xi_m - \xi_{sol})$ and $\beta_{vc} = V_{vc}(\xi_{vc} - \xi_{sol})$, respectively, where ξ_m, ξ_{vc} and ξ_{sol} are the X-ray scattering length densities of the membrane-forming block ($\xi_{PBzMA} = 10.38 \times 10^{10}$ cm^{-2}), the coronal stabiliser block ($\xi_{PSMA} = 9.24 \times 10^{10}$ cm^{-2}) and the solvent ($\xi_{sol} = 7.63 \times 10^{10}$ cm^{-2}). V_m and V_{vc} are the volumes of the membrane-forming block and the coronal stabiliser block, respectively. Using the molecular weights of the PBzMA and PSMA blocks and their respective mass densities: ρ_{PBzMA} = 1.15 g cm^{-3} and ρ_{PSMA} = 0.97 g cm^{-3}, the individual block volumes can be calculated from $V = \frac{M_{n,pol}}{N_A \rho}$, where $M_{n,pol}$ corresponds to the number-average molecular weight of the block determined by ^{1}H NMR spectroscopy. The amplitude of the membrane self-term is:

$$A_m(q) = \frac{V_{out}\varphi(qR_{out}) - V_{in}\varphi(qR_{in})}{V_{out} - V_{in}} exp\left(- \frac{q^2 \sigma_{in}^2}{2} \right) \qquad \text{(S14)}$$

where $R_{in} = R_m - \frac{1}{2}T_m$ is the inner radius of the membrane, $R_{out} = R_m + \frac{1}{2}T_m$ is the outer radius of the membrane, $V_{in} = \frac{4}{3}\pi R_{in}^3$, $V_{out} = \frac{4}{3}\pi R_{out}^3$. It should be noted that Equation (S13) differs from

the original work in which they were first described.[11] The exponent term in Equation (S14) represents a sigmoidal interface between the blocks, with a width σ_{in} accounting for a decaying scattering length density at the membrane surface. The value of σ_{in} was fixed at 2.5. The mean vesicle aggregation number, N_v, is given by:

$$N_v = (1 - x_{sol})\frac{V_{out} - V_{in}}{V_m} \tag{S15}$$

where x_{sol} is the solvent (i.e. mineral oil) volume fraction within the vesicle membrane.

A simpler expression for the corona self-term of the vesicle model than for the spherical micelle corona self-term was used due to the fact that the contribution to the scattering intensity from the corona block in this case was much less than the contribution from the membrane block. Assuming that there is no penetration of the solvophilic coronal blocks into the solvophobic membrane, the amplitude of the vesicle corona self-term is expressed as:

$$A_{vc}(q) = \Psi(qR_g)\frac{1}{2}\left[\frac{sin[q(R_{out} + R_g)]}{q(R_{out} + R_g)} + \frac{sin[q(R_{in} - R_g)]}{q(R_{in} - R_g)}\right] \tag{S16}$$

where the term outside the square brackets is the factor amplitude of the corona block polymer chain such that:

$$\Psi(qR_g) = \frac{1 - exp(-qR_g)}{(qR_g)^2} \tag{S17}$$

Again, the obtained R_g of the $PSMA_{13}$ coronal block of ~1.21 nm is comparable to the estimated value. The latter can be calculated from the total contour length of the $PSMA_{13}$ block, L_{PSMA13} = 13 × 0.255 nm = 3.315 nm (since the projected contour length per SMA monomer repeat unit is defined by two carbon bonds in an all-*trans* conformation, or 0.255 nm) and the Kuhn length of 1.53 nm [based on the known literature value for PMMA[10]] result in an approximate R_g of $(3.315 \times 1.53/6)^{1/2}$ = 0.92 nm.

It was assumed for the vesicle model that two parameters are polydisperse: the overall radius of the vesicles and the membrane thickness (R_m and T_m, respectively). They are considered to

have a Gaussian distribution and, therefore, the polydispersity function in Equation (S3) can be expressed as:

$$\Psi(r_1,r_2) = \frac{1}{\sqrt{2\pi\sigma_{Rm}{}^2}} exp\left(- \frac{(r_1 - R_m)^2}{2\sigma_{Rm}{}^2} \right) \frac{1}{\sqrt{2\pi\sigma_{Tm}{}^2}} exp\left(- \frac{(r_1 - T_m)^2}{2\sigma_{Tm}{}^2} \right) \qquad \text{(S18)}$$

where σ_{Rm} and σ_{Tm} are the standard deviations for R_m and T_m, respectively. Following Equation (S4) the number density per unit volume for the vesicle model is expressed as:

$$N = \frac{\varphi}{\int_0^\infty \int_0^\infty V(r_1,r_2)\Psi(r_1,r_2)dr_1dr_2} \qquad \text{(S19)}$$

where φ is the total *volume fraction* of copolymer in the vesicles and $V(r_1,r_2)$ is the total *volume* of copolymers in a vesicle $[V(r_1,r_2) = (V_m + V_{vc})N_v(r_1,r_2)]$. The region of the SAXS patterns which would be affected by the structure factor of concentrated vesicle dispersions were not well resolved in the performed SAXS measurements and, therefore, were excluded from the fitted pattern [i.e. only SAXS data for $q > 0.06$ nm^{-1} were used for the fitting and the structure factor in Equation (S3) was set to unity, $S(q) = 1$]. Programming tools within the Irena SAS Igor Pro macros[3] were used to implement the scattering models.

References

1. J. S. Trent, *Macromolecules*, 1984, **17**, 2930-2931.
2. M. Basham, J. Filik, M. T. Wharmby, P. C. Y. Chang, B. El Kassaby, M. Gerring, J. Aishima, K. Levik, B. C. A. Pulford, I. Sikharulidze, D. Sneddon, M. Webber, S. S. Dhesi, F. Maccherozzi, O. Svensson, S. Brockhauser, G. Naray and A. W. Ashton, *Journal of Synchrotron Radiation*, 2015, **22**, 853-858.
3. J. Ilavsky and P. R. Jemian, *Journal of Applied Crystallography*, 2009, **42**, 347-353.
4. J. S. Pedersen, *Journal of Applied Crystallography*, 2000, **33**, 637-640.
5. L. A. Fielding, J. A. Lane, M. J. Derry, O. O. Mykhaylyk and S. P. Armes, *Journal of the American Chemical Society*, 2014, **136**, 5790-5798.
6. J. S. Pedersen and M. C. Gerstenberg, *Colloids and Surfaces a-Physicochemical and Engineering Aspects*, 2003, **213**, 175-187.
7. J. S. Pedersen, C. Svaneborg, K. Almdal, I. W. Hamley and R. N. Young, *Macromolecules*, 2003, **36**, 416-433.
8. J. S. Pedersen, *Journal of Chemical Physics*, 2001, **114**, 2839-2846.
9. D. J. Kinning and E. L. Thomas, *Macromolecules*, 1984, **17**, 1712-1718.

10. L. J. Fetters, D. J. Lohsey and R. H. Colby, in *Physical Properties of Polymers Handbook*, ed. J. E. Mark, Springer, New York, 2nd edn., 2007, ch. 25, pp. 447-454.
11. J. Bang, S. M. Jain, Z. B. Li, T. P. Lodge, J. S. Pedersen, E. Kesselman and Y. Talmon, *Macromolecules*, 2006, **39**, 1199-1208.
